# Two hits are better than one: targeting both phosphatidylinositol 3-kinase and mammalian target of rapamycin as a therapeutic strategy for acute leukemia treatment

**DOI:** 10.18632/oncotarget.477

**Published:** 2012-05-04

**Authors:** Alberto M. Martelli, Francesca Chiarini, Camilla Evangelisti, Alessandra Cappellini, Francesca Buontempo, Daniela Bressanin, Milena Fini, James A. McCubrey

**Affiliations:** ^1^ Department of Human Anatomy, University of Bologna, Cellular Signalling Laboratory, Bologna, Italy; ^2^ Institute of Molecular Genetics, National Research Council-Rizzoli Orthopedic Institute, Bologna, Italy; ^3^ Department of Human, Social and Health Sciences, University of Cassino, Cassino, Italy; ^4^ Laboratory of Preclinical and Surgical Studies, Rizzoli Orthopedic Institute, Bologna, Italy; ^5^ Department of Microbiology & Immunology, Brody School of Medicine, East Carolina University, USA

**Keywords:** apoptosis, leukemia initiating cells, mRNA translation, PI3K/Akt/mTOR, targeted therapy

## Abstract

Phosphatidylinositol 3-kinase (PI3K) and mammalian target of rapamycin (mTOR) are two key components of the PI3K/Akt/mTOR signaling pathway. This signal transduction cascade regulates a wide range of physiological cell processes, that include differentiation, proliferation, apoptosis, autophagy, metabolism, motility, and exocytosis. However, constitutively active PI3K/Akt/mTOR signaling characterizes many types of tumors where it negatively influences response to therapeutic treatments. Hence, targeting PI3K/Akt/mTOR signaling with small molecule inhibitors may improve cancer patient outcome. The PI3K/Akt/mTOR signaling cascade is overactive in acute leukemias, where it correlates with enhanced drug-resistance and poor prognosis. The catalytic sites of PI3K and mTOR share a high degree of sequence homology. This feature has allowed the synthesis of ATP-competitive compounds targeting the catalytic site of both kinases. In preclinical models, dual PI3K/mTOR inhibitors displayed a much stronger cytotoxicity against acute leukemia cells than either PI3K inhibitors or allosteric mTOR inhibitors, such as rapamycin. At variance with rapamycin, dual PI3K/mTOR inhibitors targeted both mTOR complex 1 and mTOR complex 2, and inhibited the rapamycin-resistant phosphorylation of eukaryotic initiation factor 4E-binding protein 1, resulting in a marked inhibition of oncogenic protein translation. Therefore, they strongly reduced cell proliferation and induced an important apoptotic response. Here, we reviewed the evidence documenting that dual PI3K/mTOR inhibitors may represent a promising option for future targeted therapies of acute leukemia patients.

## INTRODUCTION

Acute leukemias comprise a heterogeneous group of malignant diseases that arise from immature cells of either myelogenous or lymphoid lineage. Leukemic cells are blocked at various stages of differentiation, and resistant to apoptosis. Thus, they accumulate in the bone marrow. This causes a progressive failure of normal hematopoiesis, which results in anemia, neutropenia, and thrombocytopenia. Each year, nearly 15,000 adult and pediatric patients in the United States are diagnosed with acute leukemia.

Acute myelogenous leukemia (AML) is a disorder with a median age of presentation in the late 60s. In younger patients, the incidence is two to three cases per 100,000 individuals, however in the seventh and eighth decade the incidence rises to 13 to 15 per 100,000 [1]. AML accounts for approximately 80% of all adult acute leukemias [2, 3]. Results of AML treatment have improved in younger patients who can tolerate intensified treatment strategies, however there have been very limited changes in outcome among individuals who are >60 years of age. Thus, the prognosis of AML remains poor, with an overall 5-year survival rate of 15-30%, while patients older than 60 years display an even worse prognosis (<10% survival at 5 years) [4].

Acute lymphoblastic leukemia (ALL) is caused by the uncontrolled clonal proliferation of immature lymphoid cells. In T-cell acute lymphoblastic leukemia (T-ALL), the malignant cells are derived in the thymus from T-cell progenitor cells and express immature T-cell immunophenotypic markers [5, 6]. T-cell neoplastic transformation is a complex process in which multiple lesions, involving both oncogenes and tumor suppressor genes, cooperate to alter the normal signaling pathways that regulate proliferation, differentiation, and survival of developing T-cells [7-10]. T-ALL comprises about 15% of pediatric and 25% of adult ALLs. T-ALL was associated with a very bad outcome, however the introduction of intensified polychemotherapy protocols has improved the prognosis of this disorder and current therapies can achieve 5-year relapse-free survival rates of about 75% in pediatric patients and 40-50% in adults [11, 12].

B-cell acute lymphoblastic leukemia (B-ALL) is by far the most common pediatric malignancy and comprises 85% of childhood ALL [13]. New therapeutic protocols have improved pediatric patient survival rate to approximately 80% at 5 years, however some cases still relapse and are tried by long-term side effects of therapy [14-16]. The overall prognosis of children with relapsed disease remains poor with less than 40% survival at 5 years [17]. B-ALL is a heterogeneous disorder including several subtypes with specific cellular and molecular features, that are related to clinical outcome [18]. The Philadelphia (Ph) chromosome is the most common cytogenetic anomaly associated with adult B-ALL. The Ph chromosome results from a reciprocal translocation (t) between chromosomes 9 and 22 (t[9,22][q34;q11]) [19], and results in a fusion gene on chromosome 22, i.e. the breakpoint cluster region-Abelson leukemia (Bcr-Abl) viral proto-oncogene. Bcr-Abl fusion proteins are constitutively active non-receptor tyrosine kinases that alter a myriad of intracellular signaling networks, thus contributing to leukemic cell proliferation and survival. The breakpoint may occur within one of four sites on the Bcr gene to yield three proteins of different sizes: p190, p210, and p230 [20]. The p190 Bcr-Abl fusion protein occurs in about 90% of children and between 50% and 80% of adults with Ph^+^ B-ALL. The p210 Bcr-Abl gene constitutes the rest of the Ph^+^ B-ALL population, while p230 characterizes chronic myelogenous leukemia [18]. Until recently, Ph^+^ B-ALL patients treated with conventional chemotherapy carried a very poor prognosis irrespective of their age (approximately 10% survival at 5 years). However, the outcome for patients with Ph^+^ B-ALL has improved substantially with the introduction of the tyrosine kinase inhibitor (TKI) imatinib in combination with chemotherapy [21]. Second generation TKIs (dasatinib, nilotinib) have displayed a promising activity in Ph^+^ B-ALL cases that developed resistance to imatinib due to Bcr-Abl mutations, although there are Bcr-Abl mutations, such as T315I, that are resistant to these novel TKIs [18; 22].

Since acute leukemias can still have an extremely poor outcome, at present great interest surrounds the development of novel and less toxic therapeutic strategies that may target aberrantly activated signaling networks involved in proliferation, survival, and drug-resistance of leukemic cells [23]. One such pathway is represented by the phosphatidylinositol 3-kinase (PI3K)/Akt/mammalian target of rapamycin (mTOR) signaling network. Several lines of evidence, obtained in preclinical settings of acute leukemias, have documented how this network could be targeted by small molecule protein kinase inhibitors [24-28]. Indeed, the PI3K/Akt/mTOR pathway is probably the most easily druggable signaling network in human neoplasias, and an impressive array of inhibitors, targeting critical components of this cascade, have been designed by drug companies [29]. However, optimal therapeutic strategies have yet to be identified for a successful treatment of acute leukemias. Inhibition of critical signaling nodes such as PI3K or mTOR induced cell cycle arrest, apoptosis, and lowered drug-resistance of leukemic cells [24-28]. Several phase I/II clinical trials are now underway, in which PI3K or mTOR inhibitors are being tested in leukemic patients [30-32]. In this review, we discuss the evidence documenting that dual PI3K/mTOR inhibitors could represent a promising option for future targeted therapies of patients with acute leukemias.

### LEUKEMIA INITIATING CELLS

A cancer stem cell model has been proposed for explaining malignant development, in analogy to physiological tissue renewal and differentiation. According to this model, many types of cancers, including acute leukemias, are organized hierarchically and their growth is sustained by a subpopulation of rare cancer stem cells (or cancer initiating cells) displaying asymmetric cell division, self-renewal capacity, and a limited differentiation potential [33, 34]. Cancer stem cells are mainly quiescent and intrinsically resistant to anticancer therapies. They can be serially transplanted into immunocompromised mice [typically nonobese diabetic (NOD)/severe combined immunodeficiency disease (SCID) mice] for generating tumors, and are responsible for the occurrence of metastases, drug-resistance, and relapses after induction chemotherapy or radiotherapy of the primary tumor [35, 36]. In acute leukemias, these cells are referred to as leukemia stem cells or leukemia initiating cells (LICs) [37]. LICs were first identified in AML patients by the group of John Dick in Toronto [38]. It was initially thought that in AML, LICs resided solely in the CD34^+^/CD38^−^ cell subset. Subsequent studies have highlighted that in some AML cases/subtypes, LIC activity was endowed in other cell subpopulations, displaying either a CD34^−^, or a CD34^+^/CD34^−^ phenotype [39, 40], or even a CD34^+^/CD38^+^ phenotype [41, 42].

Furthermore, the xenograft assay allowed measurement of the LIC frequency: It was found to be on the order of one per million of leukemic cells [38]. Nevertheless, this number could be deceivingly low, because it was subsequently demonstrated that leukemias of murine origin transplanted into histocompatible recipients, displayed a LIC frequency of around one to ten [43, 44]. This could be a consequence of the limited ability of human leukemic cells to adapt to grow in a more or less hostile murine microenvironment [45].

In T-ALL, multiple subpopulations endowed with LIC activity have been identified, including CD34^+^/CD4^−^, CD34^+^/CD7^−^, CD34^+^/CD7^+^, CD34^−^/CD7^+^ [46, 47]. Intriguingly, CD34 is not always a marker of stemness in T-ALL patients, especially in adult patients [48].

In B-ALL, LICs were initially described to be enriched in the CD34^+^/CD19^−^ cell subpopulation [46]. However, other groups have reported that LICs were comprised in the CD34^+^/CD19^+^ lymphoblast subset [49, 50].

The data emerging from phenotypying studies of LICs are in agreement with recent studies, performed in ALL patients, that have highlighted the genomic heterogeneity of LICs. In the first study, the authors examined pediatric B-ALL cases displaying the ETS (E-twenty six) -variant gene 6/Runt-related transcription factor-1 (ETV6/RUNX-1) fusion gene in relation to the presence of genomic copy number alterations (CNAs) [51]. By exploiting techniques capable of single cell analysis, it was documented the highly heterogeneous and diverse clonal architectures of LICs, consistently with a branching, non-linear evolutionary history of leukemia development. Accordingly, genomic CNAs occurred in various leukemic subsets in no particular order and reiteratively at various stages of the disease. Importantly, clonal architecture was dynamic and changed in the lead-up to diagnosis and in relapses. LICs xenografted in mice displayed heterogeneous genetic alterations and proliferative capacities. In the second study, where genomic CNAs were investigated in patients with Ph^+^ B-ALL, the investigators reported very similar findings [52].

Regarding T-ALL, genome-wide profiling was used to compare samples at the time of diagnosis and after engraftment into recipient mice. Compared with paired diagnosis samples, the xenografted leukemias often contained additional genomic lesions in *oncogenes* and/or tumor suppressor genes. Moreover, the xenografted leukemias appeared to arise from minor cell subsets existing in the patient at diagnosis [53]. These novel data imply that putative LICs are considerably more complex in their genomic alterations and biologic behavior than initially thought, and offer a theoretical basis for future attempts to develop effective individualized LIC-targeted therapies, that should take into account these differences [54].

The so-called side-population (SP) is thought to be enriched in cancer stem cells. SP cells actively extrude the nuclear acid-staining dye, Hoechst 33342, owing to high expression on their plasma membrane of transporters of the ATP-binding cassette (ABC) family, including ABCB1 and ABCG2, and can be easily identified by flow cytometry [55, 56]. As to acute leukemias, an enrichment of SP cells in LICs has been demonstrated in both AML [57], and T-ALL [58].

### THE PI3K/Akt/mTOR PATHWAY

PI3Ks are a family of lipid kinases that phosphorylate the 3’-OH of phosphatidylinositols. These enzymes are grouped into three classes, each with distinct substrate specificity and lipid products: I, II, and III [59]. In mammalian cells, class I PI3Ks are the best understood PI3Ks and the most widely implicated in human neoplasias [60]. For this reason, they will be the only PI3Ks highlighted here. Class I PI3Ks are further divided into two subgroups: A and B. Class IA PI3Ks contain one of three catalytic subunits (p110α, p110β, p110δ) that form heterodimers with one of the five adaptor (or regulatory) isoforms (p85α, p85β, p55α, p55γ, p50α). In general, class IA PI3Ks are activated downstream of both tyrosine kinase receptors (TKRs) and G protein-coupled receptors (GPCRs). The single class IB PI3K comprises a p110γ catalytic subunit which binds one of two related regulatory subunits, p101, and p87. Class IB PI3Ks mainly act downstream of GPCRs, however they can be stimulated also by TKRs [61]. Only class I PI3Ks have the ability to use phosphatidylinositol-4,5-bisphosphate (PtdIns 4,5P2) to generate the second messenger, phosphatidylinositol-3,4,5-trisphosphate (PtdIns 3,4,5P3).

**Figure 1:**
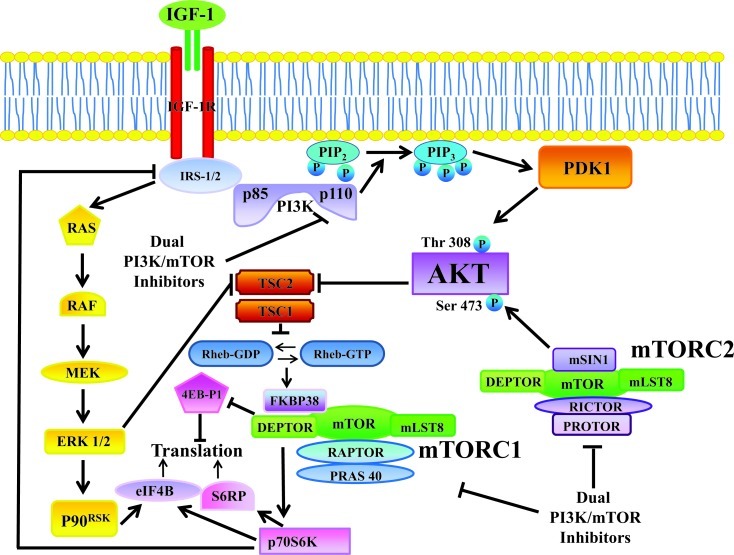
The PI3K/Akt/mTOR signaling pathway TKRs (for example, IGF-1R) stimulate class I PI3K activity. PI3K generates PtdIns 3,4,5P3 from PtdIns 4,5,P2. PtdIns 3,4,5P3 attracts to the plasma membrane PDK1 which phosphorylates Akt at Thr 308. Full Akt activation requires Ser 473 phosphorylation by mTORC2. Active Akt inhibits TSC2 activity through direct phosphorylation. TSC2 is a GTP-ase activating protein (GAP) that functions in association with TSC1 to inactivate the small G protein Rheb. Akt-driven TSC1/TSC2 complex inactivation allows Rheb to accumulate in a GTP-bound state. Rheb-GTP then upregulates the protein kinase activity of mTORC1. mTORC1 targets p70S6K, 4E-BP1, S6RP, and eIF4B which are critical for mRNA translation. However, both mTORC1 and eIF4B are targeted also by the Ras/Raf/MEK/ERK pathway. p70S6K controls activation of both PI3K and Ras through an inhibitory loop which involves IRS-1/2. Arrows indicate activating events, whereas perpendicular lines highlight inhibitory events. Deptor: DEP-domain-containing mTOR interacting protein; 4E-BP1: eukaryotic initiation factor 4E-binding protein 1; eIF4B: eukaryotic initiation factor 4B; eIF4E: eukaryotic initiation factor 4E; ERK: extracellular signal-regulated kinase; IGF-1R: insulin-like growth factor-1 receptor; IRS-1/2: insulin receptor substrate 1/2; MEK: mitogen-activated protein kinase kinase; mLST8: mammalian Lethal-with-Sec-Thirteen 8; mTOR: mammalian target of rapamycin; mTORC1: mTOR complex 1; mTORC2: mTOR complex 2; PDK1: phosphatidylinositol-dependent kinase 1; PI3K: phosphatidylinositol 3-kinase; PRAS40: proline-rich Akt substrate 40; Protor: protein observed with Rictor; PtdIns 4,5P2: PtdIns-4,5-bisphosphate; PtdIns 3,4,5P3: PtdIns-3-4,5-trisphosphate; p70S6K: p70S6 kinase; p90RSK: p90 ribosomal S6 kinase; Raptor: regulatory associated protein of mTOR; Rheb: Ras homolog enriched in brain; Rictor: rapamycin-insensitive companion of mTOR; TRK: receptor tyrosine kinase; SIN1: stress-activated protein kinase-interacting protein 1; S6RP: S6 ribosomal protein; TSC1: tuberous sclerosis 1; TSC2: tuberous sclerosis 2.

Once activated by a variety of growth factors and cytokines, class I PI3Ks initiate a cascade of events that promote cancer cell proliferation, survival, and metabolism. Akt, a 57-kDa serine/threonine kinase, is a key effector of PI3K in carcinogenesis. Akt is a member of the AGC protein kinase family and is the cellular homolog of the *v-akt* oncogene. The Akt family includes three highly conserved isoforms: Akt1/α, Akt2/β, and Akt3/γ [62]. The recruitment of inactive Akt from the cytosol to the plasma membrane, requires that the pleckstrin homology (PH) domain of Akt binds to PtdIns 3,4,5P3 synthesized at the plasma membrane by PI3K. Akt is then phosphorylated at Thr 308 by phosphatidylinositol-dependent kinase 1 (PDK1), and at Ser 473 by mTOR complex 2 (mTORC2, see later on), resulting in full activation of Akt kinase activity [63] (FIGURE 1).

Akt phosphorylates a plethora of targets [61; 64, 65] on RxRxxS/T consensus motifs [66]. Intriguingly, most of the Akt effects depend on its ability to phosphorylate proteins involved in cell cycle progression, apoptosis, mRNA translation, glycolysis, and angiogenesis, thus unlocking most, if not all, of the critical processes involved in tumorigenesis [67].

mTOR is a 289-kDa serine/threonine kinase which belongs to the phosphatidylinositol 3-kinase-related kinase (PIKK) family [68]. mTOR encompasses two functionally distinct multiprotein complexes, referred to as mTOR complex 1 (mTORC1) and mTORC2. mTORC1 is a direct downstream effector of Akt (FIGURE 1), however its activity is controlled through other signaling networks that include the Ras/Raf/mitogen-activated protein kinase kinase (MEK)/extracellular signal-regulated kinase (ERK) 1/2 signaling network (FIGURE 2), and the liver kinase B1 (LKB1)/AMP-independent protein kinase (AMPK) cascade [69, 70].

mTORC1 is characterized by the interactions between mTOR and the regulatory associated protein of mTOR (Raptor), which regulates mTOR activity and functions as a scaffold for recruiting mTORC1 substrates. mTORC1 is sensitive to rapamycin and its analogs (rapalogs) that include RAD-001, CCI-779, and AP23753. Rapamycin/rapalogs are allosteric mTORC1 inhibitors and do not target the mTOR catalytic site [71, 72]. They associate with the FK506 binding protein 12 (FKBP-12, see [73]), and, by doing so, they induce the disassembly of mTORC1, resulting in inhibition of its activity [72]. Nevertheless, there are mTORC1 outputs, such as eukaryotic initiation factor 4E-binding protein 1 (4E-BP1) phosphorylation (see later on), that are resistant to rapamycin/rapalogs, at least in some experimental models [74, 75].

mTORC2 comprises the rapamycin-insensitive companion of mTOR (Rictor) and is generally described as being insensitive to rapamycin/rapalogs. However, long-term (>24 hours) treatment of about 20% of cancer cell lines (mainly of hematopoietic lineage) with rapamycin/rapalogs resulted in mTORC2 activity inhibition [76, 77].

mTORC1 controls translation in response to growth factors/nutrients through the phosphorylation of p70S6 kinase (p70S6K) and 4E-BP1. p70S6K phosphorylates the 40S ribosomal protein, S6 (S6RP), leading to active translation of mRNAs [78]. Furthermore, p70S6K phosphorylates the eukaryotic initiation factor 4B (eIF4B) which is critically involved in translation [79, 80]. However, eIF4B is a downstream target also of MEK/ERK signaling [81] (FIGURE 1). Unphosphorylated 4E-BP1 interacts with the cap-binding protein, eukaryotic initiation factor 4E (eIF4E), and prevents the formation of the 4F translational initiation complex (eIF4F), by competing for the binding of eukaryotic initiation factor 4G (eIF4G) to eIF4E. 4E-BP1 phosphorylation by mTORC1 results in the release of the eIF4E, which then associates with eIF4G to stimulate translation initiation [82]. eIF4E is critical for translating 5'capped mRNAs, that include transcripts mainly encoding for proliferation and survival promoting proteins, such as c-Myc, cyclin-dependent kinase-2 (CDK-2), cyclin D1, signal activator and transducer of transcription-3 (STAT-3), B-cell lymphoma (Bcl) -2, Bcl-xL, survivin, myeloid cell leukemia-1 (Mcl-1), ornithine decarboxylase [76; 82, 83].

Moreover, mTORC1 represses autophagy, a lysosome-dependent degradation pathway which allows cells to recycle damaged or superfluous cytoplasmic content, such as proteins, lipids, and organelles [84]. As a consequence, cells produce metabolic precursors for macromolecular biosynthesis or ATP generation. In cancer cells, autophagy fulfils a dual role, because it can have both tumor-suppressing and tumor-promoting functions. Indeed, the autophagic machinery prevents necrosis and inflammation, that can lead to genetic instability and tumorigenesis. However, autophagy might be important for tumor progression, by providing energy through its recycling mechanism during unfavorable metabolic circumstances, that are very common in tumors [85].

The mechanisms that control mTORC2 activity have only begun to be revealed [86], however mTORC2 activation by growth factors requires PI3K, as pharmacological inhibition of PI3K decreased mTORC2 activity *in vitro* [87]. mTORC2 phosphorylates Akt on Ser 473 which enhances subsequent Akt phosphorylation on Thr 308 by PDK1.

PI3K, Akt, and mTORC1/2 are linked to each other via regulatory feedback loops, that restrain their simultaneous hyperactivation [76]. A negative regulation of Akt activity by mTORC1 is dependent on p70S6K-mediated phosphorylation of insulin receptor substrate (IRS) -1 and -2 adapter proteins, downstream of the insulin receptor (IR) and/or insulin-like growth factor-1 receptor (IGF-1R) [88-90]. IRS-1 and IRS-2 are normally required to activate class IA PI3Ks after stimulation of IR/ IGF-1R tyrosine kinase activity. When mTORC1 is active, p70S6K phosphorylates the IRS-1 and -2 proteins on Ser residues, targeting them for proteasomal degradation [91, 92]. Therefore, inhibition of mTORC1 signaling by rapamycin/rapalogs blocks this negative feedback loop and activates Akt through PI3K (FIGURE 1). Recent findings have highlighted the existence of a rapamycin-sensitive, mTORC1/p70S6K-mediated phosphorylation of Rictor at Thr 1135. This phosphorylative event exerted a negative regulatory effect on the mTORC2-dependent phosphorylation of Akt *in vivo* [93]. Thus, both mTORC1 and mTORC2 could control Akt activation.

PI3K/Akt/mTOR signaling is negatively regulated by lipid and protein phosphatases. Phosphatase and tensin homolog (PTEN) is a lipid phosphatase which removes the 3’-phosphate from PtdIns 3,4,5P3, thereby antagonizing PI3K signaling [94, 95]. Two other lipid phosphatases, Src homology domain-containing inositol phosphatase (SHIP) 1 and 2, remove the 5-phosphate from PtdIns 3,4,5P3 to yield PtdIns 3,4P2 [96]. Protein phosphatase 2A (PP2A) downregulates Akt activity directly, by dephosphorylating it at Thr 308 and several lines of evidence indicates that PP2A is a tumor suppressor [97]. Moreover, Ser 473 Akt is dephosphorylated by the two isoforms (1 and 2) of PH domain leucine-rich repeat protein phosphatase (PHLPP). Decreased PHLPP activity has been linked to specific cancer types [98, 99].

### PI3K/Akt/mTOR signaling in acute leukemias

#### AML

Activation of PI3K/Akt/mTOR signaling is a common event in AML patients. Akt phosphorylation at Ser 473, assayed by either western blotting or flow cytometry analysis of leukemic cells, could be detected in 50-80% of AML patients [100-103]. Less data are available regarding Thr 308 p-Akt. However, this amino acid residue is phosphorylated in most AML patients [104-105], and Akt phosphorylation at Thr 308, but not at Ser 473, correlated with a poorer prognosis of AML patients [104]. Interestingly, Akt phosphorylation at Ser 473 could be detected in the CD34^+^/CD38^−^/CD123^+^ leukemic cell subset which is enriched in LICs [106]. mTORC1 is activated in almost all (95%) of primary AML samples, as documented by phosphorylation of p70S6K and 4E-BP1 at Thr 389 and Thr 37/46, respectively [107, 108]. Since mTORC2 phosphorylates Akt at Ser 473, it is likely to be activated in most primary AML cells. The mechanisms leading to PI3K activation in AML cells have been partially clarified. In most cases, either p110β or p110δ (or both) PI3K could be involved in Akt phosphorylation [109-111]. Intriguingly, it has been demonstrated that IGF-1/IGF-1R signaling activates PI3K in about 70% of AML cases. Indeed, leukemic cells express functional IGF-1R, and an IGF-1 autocrine production is detectable in AML primary cells [110; 112, 113]. Accordingly, downregulation of either p110β or p110δ PI3K by RNA interference (siRNA) impaired IGF-1-stimulated Akt activation, and negatively affected proliferation and survival of AML cells [110]. Similar findings were reported in a study in which the IGF-1/IGF-1R autocrine loop was blocked by neutralizing antibodies to IGF-1R or by siRNA to IGF-1 [114]. Other TKRs that could be involved in activating PI3K in AML, include mutated (constitutively active) FMS-like tyrosine kinase 3 (FLT-3) and c-Kit. However, statistical association between FLT3-ITD (internal tandem duplication, which leads to receptor constitutive activation, see [115]) or c-Kit mutations and PI3K signaling upregulation, has not been reported in primary AML cells [116]. Nevertheless, it has been demonstrated that GRB-2-associated binder (GAB-2), which is frequently overexpressed in AML [117, 118], could mediate PI3K activation downstream of some mutant c-Kit receptors. Alterations in the activity of the PTEN and SHIP phosphatases might activate PI3K in AML, however PTEN and SHIP1 inactivating mutations/deletions are exceedingly rare in this disease [119]. Phosphorylation and decreased expression of PTEN have been reported in AML patients, however their significance remains highly controversial [120]. Recent findings have highlighted that there exists a inverse relationship between the expression levels of the B55α regulatory subunit of the PP2A phosphatase (which functions as an Akt phosphatase, see [121]) and the Thr 308 (but not Ser 473) Akt phosphorylation levels in AML primary cells. This finding suggested that B55α dephosphorylates Akt at Thr 308, but not Ser 473, in AML cells [122]. Interestingly, this study reported lower levels of the PP2A B55α regulatory subunit in AML primary cells when compared with CD34^+^ bone marrow cells from healthy donors.

Another report has documented that PP2A activity downregulation is a recurrent event in AML patients. The downregulation could be related to PP2A hyperphosphorylation at Tyr 307 in 78% of the analyzed cases. Overexpression of two endogenous PP2A inhibitors [SET (Su(var)3-9 and ‘Enhancer of zeste’ proteins, from which the acronym SET is derived), and SETBP1, a SET-binding protein] correlated with high levels of Tyr 307 phosphorylation in 55% of the cases. In some patients, the authors detected decreased expression of PP2A regulatory subunits (PPP2R1B, PPP2R5B, PPP2R5C). Thus, PP2A decreased activity in AML could be due to multiple mechanisms [123]. In any case, restoration of PP2A activity with forskolin decreased Akt phosphorylation at Thr 308 [123]. Intriguingly, the same group had previously documented that SETBP1 associated with SET and PP2A, thus forming a multi-protein complex which inhibited PP2A activity. This complex enhanced proliferation of the leukemic cells. However, the effects on Akt phosphorylation levels had not been analyzed [124].

Other potential mechanisms of PI3K/Akt activation in AML could be autocrine/paracrine secretion of vascular endothelial growth factor (VEGF) or angiopoietin [125, 126]. Indeed, an emerging theme in leukemia biology, regards the interactions between leukemic cells and cells of the bone marrow microenvironment, that include stromal cells, adipocytes, and osteoblasts [127]. These cells secrete a plethora of growth factors, cytokines, and other molecules, that could then impact on PI3K/Akt signaling in leukemic cells. The interactions between fibronectin (which is secreted by stromal cells) and the very late antigen-4 (VLA-4, expressed by leukemic cells) activated PI3K/Akt and increased drug-resistance in AML primary cells [128]. VLA-4 is formed by α4 and β1 integrin and is a key regulator of adult hematopoiesis [129]. VLA-4 increased PI3K/Akt signaling, because the integrin linked kinase (ILK), which interacts with β-integrins, is another kinase which phosphorylates Akt at Ser 473. Indeed, bone marrow-derived stromal cells induced both ILK and Akt activation in leukemic cells and QLT0267, an ILK inhibitor, blunted stromal cell-induced Ser 473 Akt phosphorylation [130].

The mechanisms leading to constitutive mTORC1 activation in primary AML blast cells are at present unclear. Several studies have demonstrated that constitutive mTORC1 activation could be PI3K-independent because: 1) in about 50% of AML primary samples, mTORC1 was activated, whereas PI3K/Akt was not [131]; 2) the p110δ PI3K selective inhibitor, IC87114, downregulated PI3K/Akt but did not suppress mTORC1 activity [116]; 3) the Src kinase Lyn was constitutively phosphorylated in some AML patients and controlled mTORC1, but not Akt, activation [132]; 4) inhibition of the IGF-1/IGF-1R autocrine loop with anti-IGF-1R antibodies inhibited Akt, but not p70S6K, phosphorylation [114]; 5) mTORC1 activity in AML samples could be decreased by inhibiting MEK/ERK signaling [133]. In this connection, it is very important to emphasize here that MEK/ERK activation is a very common event in AML patients [134].

#### T-ALL

PI3K/Akt/mTOR signaling upregulation is very common in T-ALL, being detectable in 70-85% of the patients [135], and portends a poorer prognosis [136]. Similarly to AML, multiple mechanisms could lead to PI3K/Akt/mTOR increased activity in T-ALL cells. Much attention has been devoted to PTEN, since the initial report by Ferrando and coworkers documenting that PTEN gene expression was inactivated in T-ALL cell lines and patients displaying Notch-1 activating mutations, through a repressive mechanism mediated by Hairy and Enhancer of Split homolog-1 (HES-1) [137-139]. In T-ALL cell lines, PTEN loss correlated with resistance to Notch inhibitors, raising concerns that patients with PTEN-negative disease could not respond to Notch inhibitor therapy [138]. However, it has been subsequently demonstrated that PTEN loss did not relieve primary T-ALL cells of their “addiction” to Notch-1 signaling [140]. It has been reported that PTEN downregulation could be a consequence also of miR-19 overexpression, which resulted in lower expression of several genes controlling the PI3K/Akt/mTOR cascade, including PTEN [141]. Furthermore, in a zebrafish model of T-ALL, c-Myc, which is typically overexpressed downstream of activated Notch-1 in T-ALL [142], caused PTEN mRNA downregulation [143].

Nevertheless, in most T-ALL clinical samples PTEN is expressed, but is inactivated due to phosphorylation by casein kinase 2 (CK2) and/or oxidation by reactive oxygen species (ROS), which results in overactive PI3K/Akt/mTOR signaling [135].

Mutations in PI3K, Akt, PTEN, and SHIP1 have been described in T-ALL patients. However, their frequency is very low and their functional significance with regard to PI3K/Akt/mTOR activation, has not been thoroughly assessed [144, 145].

IGF-1/IGF-1R signaling plays an important role in the activation of the PI3K/Akt/mTOR cascade in T-ALL cells, as pharmacologic inhibition or genetic deletion of IGF-1R blocked T-ALL cell proliferation and survival [146]. Interestingly, IGF-1R is a Notch-1 target gene and Notch-1 was required to maintain IGF-1R expression at high levels in T-ALL cells. Furthermore, a moderate decrease in IGF1-R signaling compromised T-ALL LIC activity [146].

In T-ALL, cytokines produced by the thymic/bone marrow microenvironment could be involved in upregulation of PI3K/Akt/mTOR signaling. These include interleukin (IL) -4 [147], and IL-7 [148, 149]. In particular, it has been recently reported that ROS produced by IL-7, are critical for activating PI3K/Akt/mTOR which then mediates proliferation and survival of T-ALL cells [150]. A source for IL-7 could be represented also by thymic epithelial cells [151]. However, increased signaling downstream of the IL-7 receptor (IL-7R) in T-ALL patients, could be a consequence of gain-of-function IL-7R mutations, which are detected in about 9% of T-ALL pediatric patients [152].

Another cytokine with the potential for activating PI3K/Akt/mTOR signaling is the CXC chemokine ligand 12 (CXCL12), referred to as SDF-1a (stromal cell-derived factor 1a), the ligand for the CXC chemokine receptor 4 (CXCR4) [153]. CXCL12 is produced by bone marrow stromal cells in T-ALL patients [154] and has been recently demonstrated to be involved in PI3K/Akt activation and drug-resistance in T-ALL cells [155].

It is not clear whether mTORC1 could be activated by signaling pathways other than PI3K/Akt in T-ALL cells. IL-7 activates MEK/ERK in T-ALL primary cells, however pharmacological inhibition of MEK/ERK did not have any negative effects on cell cycle progression and survival [148]. Thus, the pathophysiological relevance of MEK/ERK activation in T-ALL needs to be further investigated. In any case, MEK/ERK upregulation is observed in about 38% of adult T-ALL patients [156].

#### B-ALL

In Ph^+^ B-ALL, the Bcr-Abl tyrosine kinase is upstream of the PI3K/Akt/mTOR pathway [157-161]. Bcr-Abl associates with a number of proteins (c-Cbl, Shc, GRB-2, and GAB-2) that bind the p85α subunit of PI3K [162], resulting in its activation [163]. Accordingly, the Bcr-Abl inhibitor imatinib downregulated mTORC1 activity in Ph^+^ chronic myelogenous leukemia cells [164], while Ph^+^ B-ALL cell lines were hypersensitive to rapamycin [165].

PI3Ks play a key role in Bcr-Abl-dependent models of murine leukemogenesis. Indeed, it was possible to create mice that had *Pik3r1* (p85α/p55α/p50α) deleted specifically in the B-cell lineage and *Pik3r2* (p85β) deleted in all cells. As a consequence, there was decreased p190 Bcr-Abl-mediated *in vitro* colony transformation of both α^−^ and α^−^/β^−^ progenitor B-cells. Moreover, p190^+^/α^−^/β^−^ B-cells displayed a severe loss of leukemogenic potential *in vivo* [166]. However, it was found that either genetic or pharmacological (wortmannin, LY294002) inhibition of PI3K only partially reduced mTORC1 activity, as assessed by phosphorylation of S6RP in these cells. To explore the mechanism of PI3K/Akt-independent mTORC1 regulation, the authors investigated the role of two other potential mTORC1-controlling pathways: MEK/ERK and amino acid sensing. Basal ERK phosphorylation was consistently elevated in α^−^/β^−^ leukemic colony forming cells (L-CFCs) and blocked by treatment with a MEK inhibitor [166]. Nevertheless, MEK inhibition did not affect mTORC1 activity, as judged by phosphorylation of 4E-BP1, while p-S6RP levels were modestly reduced in both control and α^−^/β^−^ L-CFCs, most likely due to stimulatory effects of ERK on p70S6K [167]. When the contribution of amino acid sensing by withdrawal of leucine from the culture media was assessed, mTORC1 activity was rapidly extinguished in α^−^/β^−^ L-CFCs, as reported in other cell systems [168]. Amino acid sensing by mTORC1 was promoted by class III PI3K (hVPS34), an enzyme whose activity is sensitive to wortmannin [169]. This might explain the partial inhibition of mTORC1 signaling by wortmannin in α^−^/β^−^ L-CFCs that lack class IA PI3Ks. Therefore, residual mTORC1 activity in α^−^/β^−^ L-CFCs was MEK/ERK-independent and sustained by amino acid sensing and, perhaps, other pathways that remain to be defined [166].

There are however, Bcr-Abl-independent mechanisms of PI3K activation that resulted in imatinib resistance [170], but they have not been analyzed thoroughly.

Another reason for enhanced PI3K/Akt/mTOR signaling in Ph^+^ B-ALL is due to the fact PP2A is functionally inactivated during the blast crisis of chronic myelogenous leukemia through the inhibitory activity of SET protein, which is regulated by Bcr-Abl [171]. Reactivation of PP2A activity by FTY720 (fingolimod, a PP2A activator which has been approved as an immunomodulator for oral use in patients with multiple sclerosis [172]), led to leukemic cell growth suppression, enhanced apoptosis, impaired clonogenicity, and decreased *in vivo* leukemogenesis of imatinib- and dasatinib-sensitive and -resistant Ph^+^ B-ALL cells, as well as Ph^+^ B-ALL progenitors (CD34^+^/CD19^+^). Importantly, healthy CD34^+^ and CD34^+^/CD19^+^ bone marrow cells were unaffacted by FTY720. Moreover, pharmacologic doses of FTY720 suppressed *in vivo* Bcr-Abl-driven leukemogenesis (including leukemogenesis promoted by the T315I Bcr-Abl mutant which is resistant to imatinib and second generation TKIs) without exerting any toxicity in mice [173].

In Ph^−^ B-ALL cases, the mechanisms for PI3K/Akt/mTOR upregulation are unclear, however, they could be dependent on activation of signaling downstream of cytokine receptors, through interactions of leukemic cells with bone marrow stromal cells [174-178]. Interestingly, pediatric B-ALL patients with high expression of VLA-4 displayed an adverse outcome, which might be related to activation of PI3K/Akt/mTOR signaling [179]. Moreover, gain-of-function mutations in IL-7R have been identified in pediatric Ph^−^ B-ALL cases [180], that could account for pathway activation. Very recently, it has been shown that ETV6/RUNX1 silencing abrogated PI3K/Akt/mTOR signaling in pediatric precursor B-ALL, however, no mechanistic explanation for this phenomenon was presented [181].

### PI3K/AKT/mTOR signaling in LICs

The concept that the PI3K/Akt/mTOR signaling may serve as a therapeutic target in LICs is beginning to emerge. Over the last six years, several manuscripts have focused on the effects of PI3K/Akt/mTOR signaling activation in hematopoietic stem cells (HSCs) with regard to the development of malignant hematopoietic disorders, including acute leukemias. In a conditional PTEN knockout murine model, upon inactivation of PTEN, there was a transient increase in HSCs followed by a myeloproliferative neoplasia (MPN), and the mice subsequently developed an acute myeloid/lymphoid leukemic-like disease after 4-6 weeks [182, 183]. However, if the mice had been pretreated with rapamycin, the MPN and leukemia did not develop. The preleukemic cells that arose after conditional PTEN deletion by themselves were not able to induce acute leukemia upon transfer into SCID-recipient mice, but if the leukemic cells were derived from the PTEN^−^ conditional mice that had already developed overt leukemia, they were able to transfer leukemia to the mice, which could not be prevented by rapamycin treatment. The healthy HSCs from the PTEN conditional knockout mice repopulated the hematopoietic cell compartment of irradiated mice treated with rapamycin, indicating that it was possible to selectively eliminate preleukemic cells before the onset of an overt leukemia [183]. Consistently, Guo and coworkers [184], using a murine model of PTEN^−^ T-ALL, have documented that long-term rapamycin treatment of preleukemic mice prevented LIC formation and halted T-ALL development. Nevertheless, rapamycin did not inhibit mTORC1 signaling in the c-Kit^mid^/CD3^+^/Lin^−^ population already enriched for LICs and did not eliminate these cells. These observations may indicate that when an acute leukemia had expanded, LICs had already developed additional genetic anomalies, that could prevent the therapeutic efficacy of rapamycin, as highlighted by a previous paper documenting that multi-genetic events collaboratively contributed to PTEN^−^ T-ALL LIC formation [185].

In another study, a conditional PTEN knockout mice was crossed with a myeloid-specific Cre line in which the Cre recombinase gene was inserted into the endogenous M lysozyme locus, and therefore was under the control of myeloid-specific lysozyme promoter. Therefore, only in the myeloid linage there was a disruption of PTEN expression. In mice older than three months, an acute leukemia (resembling human acute monocytic leukemia, i.e. a subset of AML) was observed in 11 of 18 cases examined [186]. Moreover, PTEN functions as a tumor suppressor in Ph^+^ LICs [187]. Indeed, in murine models of Bcr-Abl-induced chronic myelogenous leukemia, PTEN was downregulated and PTEN deletion caused an accelerated development of the disorder. Overexpression of PTEN suppressed LICs, delayed the development of Ph^+^ B-ALL, and prolonged mice survival. These PTEN effects were mediated through Akt1. Overall, these findings supported the concept that PTEN plays a key role in the development of Ph^+^ B-ALL through PI3K/Akt/mTOR signaling [187]. As to other components of the PI3K/Akt/mTOR signaling pathway, p85α PI3K has been demonstrated to be involved in oncogenic c-Kit-induced transformation in AML, in an murine model where p85α PI3K expression was disrupted in HSCs. In contrast, p85β PI3K disruption had no functional consequences [188].

### TARGETING PI3K/Akt/mTOR SIGNALING AND ACUTE LEUKEMIAS

The fungal metabolite wortmannin and LY294002 are two well-known and isoform non-selective PI3K inhibitors. These drugs block the enzymatic activity of PI3K by different mechanisms. Wortmannin is an irreversible inhibitor (IC_50_≈2 nM) which forms a covalent bond with a conserved lysine residue involved in the phosphate-binding reaction [189], whereas LY294002 is a classical reversible, ATP-competitive PI3K modulator (IC_50_=1.40 μM) [190]. In spite of the crossover inhibition of other kinases (for example, LY294002 inhibits mTOR and CK2 with equal potency) and their unfavorable pharmaceutical properties, both wortmannin and LY294002 have served as important research tools for more than a decade in elucidating the role of PI3K in the biology of human cancers [191].

Wortmannin and LY294002 have been widely used in preclinical models of human acute leukemias, where they induced cell cycle arrest and apoptosis, as well as lowered drug-resistance to traditional chemotherapeutic drugs [100; 178; 192-195].

Given the important role played by p110δ PI3K in PI3K/Akt activation in AML, this isoform could represent a good target for pathway inhibition. IC87114, a selective p110δ PI3K inhibitor, decreased cell proliferation and survival in AML cells, and increased sensitivity to etoposide [109; 113; 196]. CAL-101 is an oral p110δ PI3K inhibitor currently undergoing clinical evaluation in patients with B-cell malignancies [197]. *In vitro*, it displayed significant cytotoxic activity in 23% of B-ALL samples tested, but only in 3% of AML samples. CAL-101 dephosphorylated Thr 308 p-Akt, and induced apoptosis in neoplastic B-cells [197]. Remarkably, CAL-101 did not affect in a substantial manner the survival of healthy B-, T-, and natural killer (NK) lymphocytes [198]. However, it was found that CAL-101 inhibited production of inflammatory cytokines, such as IL-6, IL-10, tumor necrosis factor (TNF) -α (produced by T-lymphocytes), and interferon (IFN)-γ (synthesized by NK lymphocytes). Therefore, it remains to be established whether decreased production of TNF-α and IFN-γ would impair inflammatory responses in B-ALL patients treated with CAL-101.

Rapamycin/rapalogs have been widely used both *in vitro* and *in vivo* in preclinical settings of acute leukemias, where they blocked cell proliferation and induced, sometimes, apoptosis and/or autophagy [100; 174; 199, 200] [201-203]. Moreover, several studies have highlighted that both PI3K and mTOR modulators could synergize with a wide range of drugs that are currently in use for treating acute leukemias, including chemotherapeutic drugs [108; 192, 193; 204], glucocorticoids [205-206], histone deacetylase inhibitors [207], ionizing radiation [208], proteasome inhibitors [208], all-*trans*-retinoic acid [193], and arsenic trioxide (As_2_O_3_) [209, 210].

A fundamental observation which has emerged from preclinical studies with PI3K or mTOR inhibitors, is that leukemic cells/LICs are usually more sensitive to pathway inhibition than healthy leukocytes or HSCs [108; 135; 211]. This indicates that a therapeutic window might exist which would allow the use of these drugs in humans, without serious side effects, at least at the hematopoietic system level.

Rapamycin/rapalogs have been tested in a few phase I/II studies in patients with AML, but the results have been quite disappointing [107; 212-214]. Nevertheless, there are numerous ongoing clinical trials in which allosteric mTORC1 inhibitors are being tested in patients with acute leukemias, mostly in combination with other drugs (http://clinicaltrials.gov).

### DUAL PI3K/mTOR INHIBITORS

PI-103, a morpholino quinazoline derivative, was the first disclosed ATP-competitive kinase inhibitor of mTOR which blocked the enzymatic activity of PI3K p110 isoforms, but displayed good selectivity over the rest of the human kinome [215]. PI-103 is a pan-class I PI3K inhibitor with IC_50_ values in the 2 nM (p110α PI3K) to 15 nM range (p110γ PI3K) [215-216]. It was demonstrated that PI-103, as other dual PI3K/mTOR inhibitors, inhibited both mTORC1 (IC_50_=0.02 μM) and mTORC2 (IC_50_=0.083 μM) [215; 217]. Several other similar compounds have subsequently been released, including NVP-BEZ235 [217], XL765 [218], PKI-587 [219], PF-04691502 [220], WJD008 [221], PKI-402 [222], and GNE-477 [223].

The kinase selectivity profile of these dual PI3K/mTOR modulators is consistent with the high sequence homology and identity in the ATP-catalytic cleft of these kinases. Dual PI3K/mTOR inhibitors have displayed significant, concentration-dependent cell proliferation inhibition and induction of apoptosis in a broad panel of cancer cell lines, including those harboring PI3K p110α activating mutations [224]. Overall, the *in vitro* activity of these ATP-competitive PI3K/mTOR modulators translated well in *in vivo* models of human cancer xenografted in mice. They were well tolerated and achieved tumor stasis or even regression when administered orally [191]. In spite of their high lipophilicity and limited water solubility, the pharmacological, biological and preclinical safety profiles of these dual PI3K/mTOR inhibitors supported their clinical development and a few of them (NVP-BEZ235, for example) are currently undergoing phase I/II clinical trials in cancer patients [75].

### DUAL PI3K/mTOR INHIBITORS AND ACUTE LEKEMIAS

#### PI-103

Quite a few studies have highlighted that PI-103 may be of therapeutic value in acute leukemias. As to AML, it has been documented that the inhibitor, when used alone in AML cell lines, blocked cell cycle progression in the G_1_ phase and a displayed a modest proapoptotic activity [225]. However, when combined with nutlin-3 (a small molecule inhibitor of the murine double minute (MDM) -2/p53 interactions, see [226]), PI-103 enhanced p53-dependent apoptosis in AML cell lines and primary cells expressing wild-type p53. Interestingly, PI-103 considerably enhanced proapoptotic Bax conformational change by nutlin-3 in the p53 wild-type AML cells, suggesting that PI3K/mTOR inhibition enhanced the p53-mediated mitochondrial apoptotic pathway. However, it has been highlighted that mTOR inhibition by PI-103 resulted in downregulation of p53 protein synthesis at the mRNA translational level. Interestingly, PI-103 treatment resulted in decreased expression of many pro- and antiapoptotic proteins. Levels of downstream transcriptional targets of p53 including MDM-2, p21, and Noxa were decreased. Noxa is a major p53-induced proapoptotic member of the Bcl-2 family proteins [227]. Levels of the antiapoptotic proteins Bcl-2 and survivin were decreased, whereas Puma, and Bax levels did not change significantly [225]. Cooperative dephosphorylation of the translational repressor 4E-BP1 by PI-103 and nutlin-3 indicated that mTOR inhibition and p53 activation strongly disrupted mTORC1-mediated translational control, resulting in imbalanced expression of pro- and antiapoptotic proteins and unstable mitochondrial membrane potential. PI-103 induced cleavage of caspase-3, which was enhanced by nutlin-3 cotreatment, in p53 wild-type OCI-AML-3 cells. PI-103 synergized with doxorubicin [225].

Another study has confirmed that PI-103 was essentially cytostatic for AML cell lines and blocked cells in the G_1_ phase of the cell cycle. However, in primary AML cells, PI-103 not only inhibited leukemic cell proliferation, but induced mitochondrial apoptosis, especially in a cell subset (CD34^+^/CD38^−^/CD123^+^) enriched in LICs [228]. While PI-103 affected the clonogenicity of leukemic progenitors, it did not induce apoptosis in CD34^+^ cells from healthy donors and had moderate effects on their proliferative and clonogenic properties. These findings suggested the possible existence of a therapeutic window *in vivo* if PI3K/mTOR inhibitors were used to treat leukemic patients. PI-103 displayed additive proapoptotic effects with etoposide in blast cells and in the CD34^+^/CD38^−^/CD123^+^ leukemic subpopulation [228]. More recently, it has been documented that PI-103 synergized with As_2_O_3_ in non-acute promyelocytic leukemia (APL) primary cells. As_2_O_3_ transiently upregulated PI3K/Akt/mTOR signaling in non-APL leukemic blasts and cell lines, and this increase could be blocked by PI-103 [229]. A combined As_2_O_3_/PI-103 treatment strongly synergized to kill non-APL cells and promoted their differentiation *in vitro*, as demonstrated by increased expression of CD11b and CD14 granulocytic/monocytic surface markers. Importantly, As_2_O_3_/PI-103 combined treatment led to a loss of the potential of LICs (CD34^+^/CD38^−^) to regenerate non-APL leukemia in NOD/SCID mice. The eradication of LICs was, at least in part, due to the profoundly enhanced induction of differentiation *in vivo.* Furthermore, the As_2_O_3_/PI-103 combination was almost non-toxic to hematopoietic stem cells from healthy bone marrow. It should be emphasized here that although differentiating therapy with As_2_O_3_ is very successful in APL, non-APL cases do not respond to this kind of therapy [230]. Therefore, the results reported by Hong *et al.* [229] appear very encouraging, as they indicate that As_2_O_3_ might be exploited as a new therapeutic strategy for targeting non-APL patients in combination with dual PI3K/mTOR inhibitors.

PI-103 has been studied in a preclinical setting of T-ALL, where it induced both cell cycle arrest in the G_1_ phase of the cell cycle and caspase-dependent apoptosis, at variance with rapamycin, which was mainly cytostatic. PI-103 was more effective than selective inhibitors of p110α, p110β, p110γ, and p110δ PI3K. Unlike rapamycin, PI-103 dephosphorylated Ser 473 Akt, which was indicative of mTORC2 inhibition. Remarkably, PI-103 treatment of T-ALL cell lines resulted in a marked dephosphorylation of 4E-BP1 at Thr 37/46, which was not detected with rapamycin [231]. Accordingly, PI-103 targeted protein translation more effectively than rapamycin [232]. In T-ALL cell lines, PI-103 synergized with vincristine, a chemotherapeutic drug which is employed for treating T-ALL patients [233]. Of note, PI-103 was cytotoxic not only to T-ALL cell lines, but affected the viability of T-ALL primary cells. Thus, these findings indicated that multitargeted therapy toward PI3K and mTOR might serve as an efficient treatment for those T-ALL patients that display upregulation of PI3K/Akt/mTOR signaling [231].

The antileukemic efficacy of PI-103 has been documented, both *in vitro* and *in vivo*, in Ph^+^ leukemia cells. Indeed, the drug was more effective than rapamycin at suppressing proliferation of mouse Ph^+^ pre-B-ALL co-treated with the TKI, imatinib [166]. The greater efficacy of PI-103 than rapamycin, could be explained by the fact that rapamycin induced a short time (6 hours) feedback rebound of p-Akt levels, which was not seen when cells were treated with PI-103. The findings from the murine model were then extended to human leukemias, using primary CD34^+^/CD19^+^ cells purified from peripheral blood of patients with Ph^+^ or Ph^−^ B-ALL. When their clonogenic potential was assessed, Ph^−^ B-ALL samples were resistant to imatinib, as expected. However, in each case of Ph^+^ B-ALL, the presence of PI-103 combined with imatinib suppressed colony formation to a greater extent than imatinib alone or imatinib in combination with rapamycin. Remarkably, PI-103 could potentiate the antileukemic effect of imatinib in samples derived from patients who were clinically resistant to this TKI [166]. However, it has been subsequently reported that PI-103 displayed an immunosuppressive effect in mouse *in vivo*, consisting of a reduction of the fraction of B-cells with a germinal center phenotype, a dose-dependent displacement of marginal zone B-cells, and a drop in the percentages of total splenic B- and T-cells [234]. Therefore, the potential immunosuppressive effects of dual PI3K/mTOR inhibitors should be carefully evaluated in future studies.

#### NVP-BEZ235

PI-103 displayed a remarkable efficacy *in vitro* and *in vivo* in preclinical settings of human and mouse neoplasias, however, its pharmacological features were found to be unfavourable, so any further development for its use in humans has been precluded [235]. NVP-BEZ235 is an orally bioavailable drug containing a tricyclic imidazo quinoline core scaffold, which has entered phase I/II clinical trials for solid tumors [217]. Recently, its efficacy has been tested against human AML cell lines and primary samples [236]. NVP-BEZ235 inhibited PI3K, mTORC1, and mTORC2 signaling, as demonstrated by the dephosphorylation of paxillin, a read-out for mTORC2 activity [237]. Furthermore, NVP-BEZ235 fully inhibited the rapamycin-resistant phosphorylation of 4E-BP1, causing a marked inhibition of protein translation in AML cells. This resulted in reduced levels of the expression of three highly oncogenic proteins known to be regulated at the translation initiation level, i.e. c-Myc, cyclin D1, and Bcl-xL [238]. Hence, NVP-BEZ235 reduced the proliferation and induced an important apoptotic response in AML cells without affecting healthy CD34^+^ cell survival. Moreover, it affected the clonogenic activity of leukemic, but not healthy, CD34^+^ cells [236].

NVP-BEZ235 was cytotoxic to a panel of T-ALL cell lines and caused cell cycle arrest, apoptosis, and autophagy. Western blots documented a dose- and time-dependent dephosphorylation of Akt and mTORC1 downstream targets in response to NVP-BEZ235 [239]. Remarkably, NVP-BEZ235 targeted the SP of both T-ALL cell lines and patient lymphoblasts, which might correspond to LICs, and synergized with several chemotherapeutic agents (cyclophosphamide, cytarabine, dexamethasone) currently used for treating T-ALL patients. NVP-BEZ235 reduced chemoresistance to vincristine induced in Jurkat cells by coculturing with MS-5 stromal cells, that mimic the bone marrow microenvironment [240]. NVP-BEZ235 was cytotoxic to T-ALL patient lymphoblasts displaying pathway activation, where the drug dephosphorylated 4E-BP1, at variance with rapamycin. Taken together, these findings indicated that longitudinal inhibition at two nodes of the PI3K/Akt/mTOR network with NVP-BEZ235, either alone or in combination with chemotherapeutic drugs, may be an efficient treatment of those T-ALL cases that have aberrant upregulation of this signaling pathway [239]. Antiproliferative effects of NVP-BEZ235 in ALL cells were associated with reduced levels of cyclin-dependent kinase 4 and cyclin D3 [241].

NVP-BEZ235 has been evaluated in a mouse model consisting of BA/F3 cells overexpressing either wild-type Bcr-Abl or its imatinib-resistant mutant forms (E255K and T315I) [242]. NVP-BEZ235 inhibited proliferation of both wild-type and mutant Bcr-Abl overexpressing cells, whereas parental Ba/F3 cells were much less sensitive. The drug induced apoptosis, and inhibited both mTORC1 and mTORC2 signaling. Remarkably, the drug displayed cytotoxic activity *in vivo* against leukemic cells expressing the E255K and T315I Bcr-Abl mutant forms [242]. However, in this experimental model NVP-BEZ235 induced an overactivation of MEK/ERK signaling, most likely due to the well-known compensatory feedback mechanism that involves p70S6K [243].

#### NVP-BAG956

This drug was originally described as a dual PI3K/PDK1 inhibitor [244], however it was subsequently disclosed that it inhibited mTOR [245]. NVP-BAG956 blocked Akt phosphorylation induced by Bcr-Abl and caused apoptosis of Bcr-Abl-expressing cell lines and patient bone marrow cells at concentrations that inhibited PI3K signaling. Enhancement of the inhibitory effects of the TKIs, imatinib and nilotinib, by NVP-BAG956 was demonstrated against Bcr-Abl expressing cells both *in vitro* and *in vivo*. It was documented that NVP-BAG956 was effective against mutant FLT3-expressing cell lines and AML patient bone marrow cells. Enhancement of the inhibitory effects of the TKI, PKC412, by NVP-BAG956 was demonstrated against mutant FLT3-expressing cells. Finally, NVP-BAG956 and rapamycin/RAD001 were shown to combine in a non-antagonistic fashion against Bcr-Abl- and mutant FLT3-expressing cells both *in vitro* and *in vivo* [244].

## CONCLUSIONS AND FUTURE PERSPECTIVES

Several lines of evidence have documented that PI3K and mTOR are key nodes in the PI3K/Akt/mTOR signaling cascade, which is by far the most commonly upregulated pathway in human cancers, including acute leukemias [246-253]. Several major pharmaceutical companies are currently testing the hypothesis that dual PI3K/mTOR inhibitors, used either alone or in combination with other drugs, will be able to overcome the limited clinical responses that have been observed with PI3K-selective inhibitors or rapamycin/rapalogs [254-256]. The findings reviewed in this article suggest that there is a strong rationale for targeting both PI3K and mTOR in acute leukemias. Indeed, it should be considered that mTORC1 could be activated by signaling pathways other than PI3K/Akt/mTOR (MEK/ERK, for example), hence mTORC1 activity could escape the inhibition of signals downstream of PI3K, as reported in AML [116]. Dual PI3K/mTOR inhibitors target both mTORC1 and mTORC2, and are powerful suppressors of cell cycle progression and inducers of apoptosis, most likely because they efficiently block translation of short-lived oncogenetic proteins [257, 258]. Moreover, in most experimental models dual PI3K/mTOR inhibitors did not cause the Akt hyperactivation which is often detected when rapamycin/rapalogs are employed in anti-cancer therapies [259]. However, there are two reports that have documented that NVP-BEZ235 resulted in MEK/ERK overactivation in Bcr-Abl-expressing murine cells [242], and in human glioblastoma-like stem cells [260]. Indeed, it is now clear that PI3K/Akt/mTORC1 and MEK/ERK pathways repress each other through a p70S6K-mediated negative feedback loop [260]. Therefore, this is an issue that will require further investigation in models of human acute leukemias, as it could indicate the need for combining dual PI3K/mTOR inhibitors with MEK inhibitors. Furthermore, dual PI3K/mTOR inhibitors are pan-PI3K p110 inhibitors, and this could represent an additional advantage over selective PI3K isoform inhibitors, as in both AML and T-ALL more than one PI3K p110 isoform is usually active.

PI3K has numerous roles in cell survival, differentiation, metabolism and migration, some of which are independent of mTOR [60; 261]. Therefore, it remains to be determined whether simultaneous inhibition of PI3K and mTOR will provide an acceptable therapeutic window in humans. For example, we do not know the *in vivo* toxicity of dual PI3K/mTOR inhibitors towards the immune system and hematopoiesis. Could it be possible to specifically target PI3K/Akt/mTOR signaling in LICs, without affecting the functions of HSCs? Indeed, evidence suggests that this pathway is important for the biology of normal stem cells, including HSCs [32; 69; 262-264]. NVP-BEZ235 did not display detrimental effects *in vitro* on healthy T-lymphocytes proliferation or the clonogenic activity of normal CD34^+^ hematopoietic progenitor cells [236; 239]. Nevertheless, results from Fruman’s laboratory seem to indicate that PI-103 suppressed hematopoietic colony formation and B-cell proliferation more strongly than an mTORC1/mTORC2 kinase inhibitor [234]. A recent report documented that PI-103 lowered lymphocyte numbers *in vivo* and suppressed immune rejection of melanoma xenografts [265]. It remains to be established whether the different effects displayed by NVP-BEZ235 and PI-103 on healthy lymphocytes and hematopoietic cells were related to the different experimental models or reflected the different chemical structure of these drugs.

However, there are preliminary data indicating that there exist subtle differences in how HSCs and LICs utilize the same signaling pathways. This has been demonstrated in LICs treated with rapamycin [183]. In this case, the drug did not affect HSCs, whereas it was cytotoxic to LICs. These observations have provided the proof-of-principle that functional differences in signaling pathways between neoplastic and healthy stem cells could be identified and exploited for targeted therapy. Ultimately, the most efficacious use of any kind of signal transduction modulator will be in the context of personalized medicine [266]. Indeed, if LICs are to be successfully targeted in clinical settings of acute leukemias, this will require a comprehensive understanding of how signaling pathways function in cancer cells when compared to their healthy counterpart. Therefore, a major challenge in the clinical use of PI3K/Akt/mTOR pathway inhibitors will be the identification of patients who will likely respond to the treatment. For example, it has been recently documented that high levels of the cell cycle progression inhibitor, p27^Kip1^, sensitized pituitary adenomas to NVP-BEZ235 [267], whereas amplification of either c-Myc or eIF4E rendered breast cancer cells resistant to NVP-BEZ235 [268]. Intriguingly, c-Myc is frequently overexpressed in T-ALL [269], whereas increased expression levels of eIF4E have been recently reported in AML [270].

Thus, a thorough evaluation of cross-talks between signaling pathways aberrantly activated in LICs coupled to the identification of specific PI3K/Akt/mTOR substrates and of their roles in the quiescence, proliferation, survival, and drug-resistance of LICs as compared with HSCs, could provide the rationale for developing personalized pharmacological treatments, based on dual PI3K/mTOR inhibitors, aimed to acute leukemia eradication.

Therefore, also acute leukemias could be added to the growing list of disorders where PI3K and mTOR inhibition could be beneficial to patients [271-276].

## References

[1] Burnett A, Wetzler M, Lowenberg B (2011). Therapeutic advances in acute myeloid leukemia. J Clin Oncol.

[2] Lowenberg B, Downing JR, Burnett A (1999). Acute myeloid leukemia. N Engl J Med.

[3] Dores GM, Devesa SS, Curtis RE, Linet MS, Morton LM (2012). Acute leukemia incidence and patient survival among children and adults in the United States, 2001-2007. Blood.

[4] Theilgaard-Monch K, Boultwood J, Ferrari S, Giannopoulos K, Hernandez-Rivas JM, Kohlmann A, Morgan M, Porse B, Tagliafico E, Zwaan CM, Wainscoat J, Van den Heuvel-Eibrink MM, Mills K, Bullinger L (2011). Gene expression profiling in MDS and AML: potential and future avenues. Leukemia.

[5] Zhao WL (2010). Targeted therapy in T-cell malignancies: dysregulation of the cellular signaling pathways. Leukemia.

[6] Kox C, Zimmermann M, Stanulla M, Leible S, Schrappe M, Ludwig WD, Koehler R, Tolle G, Bandapalli OR, Breit S, Muckenthaler MU, Kulozik AE (2010). The favorable effect of activating NOTCH1 receptor mutations on long-term outcome in T-ALL patients treated on the ALL-BFM 2000 protocol can be separated from FBXW7 loss of function. Leukemia.

[7] Zuurbier L, Homminga I, Calvert V, te Winkel ML, Buijs-Gladdines JG, Kooi C, Smits WK, Sonneveld E, Veerman AJ, Kamps WA, Horstmann M, Petricoin EF, Pieters R, Meijerink JP (2010). NOTCH1 and/or FBXW7 mutations predict for initial good prednisone response but not for improved outcome in pediatric T-cell acute lymphoblastic leukemia patients treated on DCOG or COALL protocols. Leukemia.

[8] Clappier E, Collette S, Grardel N, Girard S, Suarez L, Brunie G, Kaltenbach S, Yakouben K, Mazingue F, Robert A, Boutard P, Plantaz D, Rohrlich P, van Vlierberghe P, Preudhomme C, Otten J, Speleman F, Dastugue N, Suciu S, Benoit Y, Bertrand Y, Cave H (2010). NOTCH1 and FBXW7 mutations have a favorable impact on early response to treatment, but not on outcome, in children with T-cell acute lymphoblastic leukemia (T-ALL) treated on EORTC trials 58881 and 58951. Leukemia.

[9] Renneville A, Kaltenbach S, Clappier E, Collette S, Micol JB, Nelken B, Lepelley P, Dastugue N, Benoit Y, Bertrand Y, Preudhomme C, Cave H (2010). Wilms tumor 1 (WT1) gene mutations in pediatric T-cell malignancies. Leukemia.

[10] Yu L, Slovak ML, Mannoor K, Chen C, Hunger SP, Carroll AJ, Schultz RA, Shaffer LG, Ballif BC, Ning Y (2011). Microarray detection of multiple recurring submicroscopic chromosomal aberrations in pediatric T-cell acute lymphoblastic leukemia. Leukemia.

[11] Pui CH, Robison LL, Look AT (2008). Acute lymphoblastic leukaemia. Lancet.

[12] Koch U, Radtke F (2011). Notch in T-ALL: new players in a complex disease. Trends Immunol.

[13] Cocco C, Airoldi I (2010). Cytokines and microRNA in pediatric B-acute lymphoblastic leukemia. Cytokine Growth Factor. Rev.

[14] Salzer WL, Devidas M, Carroll WL, Winick N, Pullen J, Hunger SP, Camitta BA (2010). Long-term results of the pediatric oncology group studies for childhood acute lymphoblastic leukemia 1984-2001: a report from the children’s oncology group. Leukemia.

[15] Moricke A, Zimmermann M, Reiter A, Henze G, Schrauder A, Gadner H, Ludwig WD, Ritter J, Harbott J, Mann G, Klingebiel T, Zintl F, Niemeyer C, Kremens B, Niggli F, Niethammer D, Welte K, Stanulla M, Odenwald E, Riehm H, Schrappe M (2010). Long-term results of five consecutive trials in childhood acute lymphoblastic leukemia performed by the ALL-BFM study group from 1981 to 2000. Leukemia.

[16] Bassan R, Hoelzer D (2011). Modern therapy of acute lymphoblastic leukemia. J Clin Oncol.

[17] Pui CH, Evans WE (2006). Treatment of acute lymphoblastic leukemia. N Engl J Med.

[18] Lee HJ, Thompson JE, Wang ES, Wetzler M (2011). Philadelphia chromosome-positive acute lymphoblastic leukemia: current treatment and future perspectives. Cancer.

[19] Rowley JD (1973). Letter: A new consistent chromosomal abnormality in chronic myelogenous leukaemia identified by quinacrine fluorescence and Giemsa staining. Nature.

[20] Melo JV (1996). The diversity of BCR-ABL fusion proteins and their relationship to leukemia phenotype. Blood.

[21] Vignetti M, Fazi P, Cimino G, Martinelli G, Di Raimondo F, Ferrara F, Meloni G, Ambrosetti A, Quarta G, Pagano L, Rege-Cambrin G, Elia L, Bertieri R, Annino L, Foa R, Baccarani M, Mandelli F (2007). Imatinib plus steroids induces complete remissions and prolonged survival in elderly Philadelphia chromosome-positive patients with acute lymphoblastic leukemia without additional chemotherapy: results of the Gruppo Italiano Malattie Ematologiche dell’Adulto (GIMEMA) LAL0201-B protocol. Blood.

[22] Ottmann O, Dombret H, Martinelli G, Simonsson B, Guilhot F, Larson RA, Rege-Cambrin G, Radich J, Hochhaus A, Apanovitch AM, Gollerkeri A, Coutre S (2007). Dasatinib induces rapid hematologic and cytogenetic responses in adult patients with Philadelphia chromosome positive acute lymphoblastic leukemia with resistance or intolerance to imatinib: interim results of a phase 2 study. Blood.

[23] Steelman LS, Franklin RA, Abrams SL, Chappell W, Kempf CR, Basecke J, Stivala F, Donia M, Fagone P, Nicoletti F, Libra M, Ruvolo P, Ruvolo V, Evangelisti C, Martelli AM, McCubrey JA (2011). Roles of the Ras/Raf/MEK/ERK pathway in leukemia therapy. Leukemia.

[24] Martelli AM, Evangelisti C, Chiarini F, Grimaldi C, Manzoli L, McCubrey JA (2009). Targeting the PI3K/AKT/mTOR signaling network in acute myelogenous leukemia. Expert Opin Investig Drugs.

[25] Crazzolara R, Bendall L (2009). Emerging treatments in acute lymphoblastic leukemia. Curr Cancer Drug Targets.

[26] Teachey DT, Grupp SA, Brown VI (2009). Mammalian target of rapamycin inhibitors and their potential role in therapy in leukaemia and other haematological malignancies. Br J Haematol.

[27] Khwaja A (2010). PI3K as a target for therapy in haematological malignancies. Curr Topics Microbiol Immunol.

[28] Martelli AM, Evangelisti C, Chiarini F, McCubrey JA (2010). The phosphatidylinositol 3-kinase/Akt/mTOR signaling network as a therapeutic target in acute myelogenous leukemia patients. Oncotarget.

[29] Emerling BM, Akcakanat A (2011). Targeting PI3K/mTOR Signaling in Cancer. Cancer Res.

[30] Chapuis N, Tamburini J, Green AS, Willems L, Bardet V, Park S, Lacombe C, Mayeux P, Bouscary D (2010). Perspectives on inhibiting mTOR as a future treatment strategy for hematological malignancies. Leukemia.

[31] Martelli AM, Evangelisti C, Chappell W, Abrams SL, Basecke J, Stivala F, Donia M, Fagone P, Nicoletti F, Libra M, Ruvolo V, Ruvolo P, Kempf CR, Steelman LS, McCubrey JA (2011). Targeting the translational apparatus to improve leukemia therapy: roles of the PI3K/PTEN/Akt/mTOR pathway. Leukemia.

[32] Polak R, Buitenhuis M (2011). The PI3K/PKB signaling module as key regulator of hematopoiesis: implications for therapeutic strategies in leukemia. Blood.

[33] Pietras A (2011). Cancer stem cells in tumor heterogeneity. Adv Cancer Res.

[34] Clevers H (2011). The cancer stem cell: premises, promises and challenges. Nat Med.

[35] Martelli AM, Evangelisti C, Follo MY, Ramazzotti G, Fini M, Giardino R, Manzoli L, McCubrey JA, Cocco L (2011). Targeting the phosphatidylinositol 3-kinase/Akt/mammalian target of rapamycin signaling network in cancer stem cells. Curr Med Chem.

[36] Cheng L, Alexander R, Zhang S, Pan CX, MacLennan GT, Lopez-Beltran A, Montironi R (2011). The clinical and therapeutic implications of cancer stem cell biology. Expert Rev Anticancer Ther.

[37] Buss EC, Ho AD (2011). Leukemia stem cells. Int J Cancer.

[38] Lapidot T, Sirard C, Vormoor J, Murdoch B, Hoang T, Caceres-Cortes J, Minden M, Paterson B, Caligiuri MA, Dick JE (1994). A cell initiating human acute myeloid leukaemia after transplantation into SCID mice. Nature.

[39] Taussig DC, Vargaftig J, Miraki-Moud F, Griessinger E, Sharrock K, Luke T, Lillington D, Oakervee H, Cavenagh J, Agrawal SG, Lister TA, Gribben JG, Bonnet D (2010). Leukemia-initiating cells from some acute myeloid leukemia patients with mutated nucleophosmin reside in the CD34(-) fraction. Blood.

[40] Martelli MP, Pettirossi V, Thiede C, Bonifacio E, Mezzasoma F, Cecchini D, Pacini R, Tabarrini A, Ciurnelli R, Gionfriddo I, Manes N, Rossi R, Giunchi L, Oelschlagel U, Brunetti L, Gemei M, Delia M, Specchia G, Liso A, Di Ianni M, Di Raimondo F, Falzetti F, Del Vecchio L, Martelli MF, Falini B (2010). CD34+ cells from AML with mutated NPM1 harbor cytoplasmic mutated nucleophosmin and generate leukemia in immunocompromised mice. Blood.

[41] Taussig DC, Miraki-Moud F, Anjos-Afonso F, Pearce DJ, Allen K, Ridler C, Lillington D, Oakervee H, Cavenagh J, Agrawal SG, Lister TA, Gribben JG, Bonnet D (2008). Anti-CD38 antibody-mediated clearance of human repopulating cells masks the heterogeneity of leukemia-initiating cells. Blood.

[42] Eppert K, Takenaka K, Lechman ER, Waldron L, Nilsson B, van Galen P, Metzeler KH, Poeppl A, Ling V, Beyene J, Canty AJ, Danska JS, Bohlander SK, Buske C, Minden MD, Golub TR, Jurisica I, Ebert BL, Dick JE (2010). Stem cell gene expression programs influence clinical outcome in human leukemia. Nat Med.

[43] Kelly PN, Dakic A, Adams JM, Nutt SL, Strasser A (2007). Tumor growth need not be driven by rare cancer stem cells. Science.

[44] Williams RT, den Besten W, Sherr CJ (2007). Cytokine-dependent imatinib resistance in mouse BCR-ABL+, Arf-null lymphoblastic leukemia. Genes Dev.

[45] Meyer LH, Debatin KM (2011). Diversity of Human Leukemia Xenograft Mouse Models: Implications for Disease Biology. Cancer Res.

[46] Cox CV, Martin HM, Kearns PR, Virgo P, Evely RS, Blair A (2007). Characterization of a progenitor cell population in childhood T-cell acute lymphoblastic leukemia. Blood.

[47] Gerby B, Clappier E, Armstrong F, Deswarte C, Calvo J, Poglio S, Soulier J, Boissel N, Leblanc T, Baruchel A, Landman-Parker J, Romeo PH, Ballerini P, Pflumio F (2011). Expression of CD34 and CD7 on human T-cell acute lymphoblastic leukemia discriminates functionally heterogeneous cell populations. Leukemia.

[48] Chiu PP, Jiang H, Dick JE (2010). Leukemia-initiating cells in human T-lymphoblastic leukemia exhibit glucocorticoid resistance. Blood.

[49] Hong D, Gupta R, Ancliff P, Atzberger A, Brown J, Soneji S, Green J, Colman S, Piacibello W, Buckle V, Tsuzuki S, Greaves M, Enver T (2008). Initiating and cancer-propagating cells in TEL-AML1-associated childhood leukemia. Science.

[50] Kong Y, Yoshida S, Saito Y, Doi T, Nagatoshi Y, Fukata M, Saito N, Yang SM, Iwamoto C, Okamura J, Liu KY, Huang XJ, Lu DP, Shultz LD, Harada M, Ishikawa F (2008). CD34+CD38+CD19+ as well as CD34+CD38-CD19+ cells are leukemia-initiating cells with self-renewal capacity in human B-precursor ALL. Leukemia.

[51] Anderson K, Lutz C, van Delft FW, Bateman CM, Guo Y, Colman SM, Kempski H, Moorman AV, Titley I, Swansbury J, Kearney L, Enver T, Greaves M (2011). Genetic variegation of clonal architecture and propagating cells in leukaemia. Nature.

[52] Notta F, Mullighan CG, Wang JC, Poeppl A, Doulatov S, Phillips LA, Ma J, Minden MD, Downing JR, Dick JE (2011). Evolution of human BCR-ABL1 lymphoblastic leukaemia-initiating cells. Nature.

[53] Clappier E, Gerby B, Sigaux F, Delord M, Touzri F, Hernandez L, Ballerini P, Baruchel A, Pflumio F, Soulier J (2011). Clonal selection in xenografted human T cell acute lymphoblastic leukemia recapitulates gain of malignancy at relapse. J Exp Med.

[54] Rasheed ZA, Kowalski J, Smith BD, Matsui W (2011). Concise review: Emerging concepts in clinical targeting of cancer stem cells. Stem Cells.

[55] Goodell MA, Brose K, Paradis G, Conner AS, Mulligan RC (1996). Isolation and functional properties of murine hematopoietic stem cells that are replicating in vivo. J Exp Med.

[56] Goodell MA, Rosenzweig M, Kim H, Marks DF, DeMaria M, Paradis G, Grupp SA, Sieff CA, Mulligan RC, Johnson RP (1997). Dye efflux studies suggest that hematopoietic stem cells expressing low or undetectable levels of CD34 antigen exist in multiple species. Nat Med.

[57] Moshaver B, van Rhenen A, Kelder A, van der Pol M, Terwijn M, Bachas C, Westra AH, Ossenkoppele GJ, Zweegman S, Schuurhuis GJ (2008). Identification of a small subpopulation of candidate leukemia-initiating cells in the side population of patients with acute myeloid leukemia. Stem Cells.

[58] Yamazaki J, Mizukami T, Takizawa K, Kuramitsu M, Momose H, Masumi A, Ami Y, Hasegawa H, Hall WW, Tsujimoto H, Hamaguchi I, Yamaguchi K (2009). Identification of cancer stem cells in a Tax-transgenic (Tax-Tg) mouse model of adult T-cell leukemia/lymphoma. Blood.

[59] Cantley LC (2002). The phosphoinositide 3-kinase pathway. Science.

[60] Engelman JA, Luo J, Cantley LC (2006). The evolution of phosphatidylinositol 3-kinases as regulators of growth and metabolism. Nat Rev Genet.

[61] Franke TF (2008). PI3K/Akt: getting it right matters. Oncogene.

[62] Brazil DP, Yang ZZ, Hemmings BA (2004). Advances in protein kinase B signalling: AKTion on multiple fronts. Trends Biochem Sci.

[63] Georgescu MM (2010). PTEN Tumor Suppressor Network in PI3K-Akt Pathway Control. Genes Cancer.

[64] Brazil DP, Park J, Hemmings BA (2002). PKB binding proteins. Getting in on the Akt. Cell.

[65] Manning BD, Cantley LC (2007). AKT/PKB signaling: navigating downstream. Cell.

[66] Alessi DR, Andjelkovic M, Caudwell B, Cron P, Morrice N, Cohen P, Hemmings BA (1996). Mechanism of activation of protein kinase B by insulin and IGF-1. EMBO J.

[67] Hanahan D, Weinberg RA (2000). The hallmarks of cancer. Cell.

[68] Memmott RM, Dennis PA (2009). Akt-dependent and -independent mechanisms of mTOR regulation in cancer. Cell Signal.

[69] Martelli AM, Evangelisti C, Chiarini F, Grimaldi C, Cappellini A, Ognibene A, McCubrey JA (2010). The emerging role of the phosphatidylinositol 3-kinase/Akt/mammalian target of rapamycin signaling network in normal myelopoiesis and leukemogenesis. Biochim Biophys Acta.

[70] Inoki K, Kim J, Guan KL (2012). AMPK and mTOR in Cellular Energy Homeostasis and Drug Targets. Annu Rev Pharmacol Toxicol.

[71] Kim DH, Sarbassov DD, Ali SM, King JE, Latek RR, Erdjument-Bromage H, Tempst P, Sabatini DM (2002). mTOR interacts with raptor to form a nutrient-sensitive complex that signals to the cell growth machinery. Cell.

[72] Oshiro N, Yoshino K, Hidayat S, Tokunaga C, Hara K, Eguchi S, Avruch J, Yonezawa K (2004). Dissociation of raptor from mTOR is a mechanism of rapamycin-induced inhibition of mTOR function. Genes Cells.

[73] Bai X, Ma D, Liu A, Shen X, Wang QJ, Liu Y, Jiang Y (2007). Rheb activates mTOR by antagonizing its endogenous inhibitor, FKBP38. Science.

[74] Shor B, Gibbons JJ, Abraham RT, Yu K (2009). Targeting mTOR globally in cancer: thinking beyond rapamycin. Cell Cycle.

[75] Garcia-Echeverria C (2010). Allosteric and ATP-competitive kinase inhibitors of mTOR for cancer treatment. Bioorg Med Chem Lett.

[76] Dunlop EA, Tee AR (2009). Mammalian target of rapamycin complex 1: signalling inputs, substrates and feedback mechanisms. Cell Signal.

[77] Rosner M, Hengstschlager M (2008). Cytoplasmic and nuclear distribution of the protein complexes mTORC1 and mTORC2: rapamycin triggers dephosphorylation and delocalization of the mTORC2 components rictor and sin1. Hum Mol Genet.

[78] Browne GJ, Proud CG (2004). A novel mTOR-regulated phosphorylation site in elongation factor 2 kinase modulates the activity of the kinase and its binding to calmodulin. Mol Cell Biol.

[79] Ma XM, Blenis J (2009). Molecular mechanisms of mTOR-mediated translational control. Nat Rev Mol Cell Biol.

[80] Shahbazian D, Parsyan A, Petroulakis E, Topisirovic I, Martineau Y, Gibbs BF, Svitkin Y, Sonenberg N (2010). Control of cell survival and proliferation by mammalian eukaryotic initiation factor 4B. Mol Cell Biol.

[81] van Gorp AG, van der Vos KE, Brenkman AB, Bremer A, van den Broek N, Zwartkruis F, Hershey JW, Burgering BM, Calkhoven CF, Coffer PJ (2009). AGC kinases regulate phosphorylation and activation of eukaryotic translation initiation factor 4B. Oncogene.

[82] Blagden SP, Willis AE (2011). The biological and therapeutic relevance of mRNA translation in cancer. Nat Rev Clin Oncol.

[83] Mamane Y, Petroulakis E, LeBacquer O, Sonenberg N (2006). mTOR, translation initiation and cancer. Oncogene.

[84] Chen S, Rehman SK, Zhang W, Wen A, Yao L, Zhang J (2010). Autophagy is a therapeutic target in anticancer drug resistance. Biochim Biophys Acta.

[85] Janku F, McConkey DJ, Hong DS, Kurzrock R (2011). Autophagy as a target for anticancer therapy. Nat Rev Clin Oncol.

[86] Sparks CA, Guertin DA (2010). Targeting mTOR: prospects for mTOR complex 2 inhibitors in cancer therapy. Oncogene.

[87] Huang J, Dibble CC, Matsuzaki M, Manning BD (2008). The TSC1-TSC2 complex is required for proper activation of mTOR complex 2. Mol Cell Biol.

[88] Shah OJ, Wang Z, Hunter T (2004). Inappropriate activation of the TSC/Rheb/mTOR/S6K cassette induces IRS1/2 depletion, insulin resistance, and cell survival deficiencies. Curr Biol.

[89] Lang SA, Hackl C, Moser C, Fichtner-Feigl S, Koehl GE, Schlitt HJ, Geissler EK, Stoeltzing O (2010). Implication of RICTOR in the mTOR inhibitor-mediated induction of insulin-like growth factor-I receptor (IGF-IR) and human epidermal growth factor receptor-2 (Her2) expression in gastrointestinal cancer cells. Biochim Biophys Acta.

[90] Bhaskar PT, Hay N (2007). The two TORCs and Akt. Dev Cell.

[91] Xu X, Sarikas A, Dias-Santagata DC, Dolios G, Lafontant PJ, Tsai SC, Zhu W, Nakajima H, Nakajima HO, Field LJ, Wang R, Pan ZQ (2008). The CUL7 E3 ubiquitin ligase targets insulin receptor substrate 1 for ubiquitin-dependent degradation. Mol Cell.

[92] Sriburi R, Jackowski S, Mori K, Brewer JW (2004). XBP1: a link between the unfolded protein response, lipid biosynthesis, and biogenesis of the endoplasmic reticulum. J Cell Biol.

[93] Dibble CC, Asara JM, Manning BD (2009). Characterization of Rictor phosphorylation sites reveals direct regulation of mTOR complex 2 by S6K1. Mol Cell Biol.

[94] Keniry M, Parsons R (2008). The role of PTEN signaling perturbations in cancer and in targeted therapy. Oncogene.

[95] Stiles BL (2009). Phosphatase and tensin homologue deleted on chromosome 10: extending its PTENtacles. Int J Biochem Cell Biol.

[96] Kalesnikoff J, Sly LM, Hughes MR, Buchse T, Rauh MJ, Cao LP, Lam V, Mui A, Huber M, Krystal G (2003). The role of SHIP in cytokine-induced signaling. Rev Physiol Biochem Pharmacol.

[97] Eichhorn PJ, Creyghton MP, Bernards R (2009). Protein phosphatase 2A regulatory subunits and cancer. Biochim Biophys Acta.

[98] Brognard J, Newton AC (2008). PHLiPPing the switch on Akt and protein kinase C signaling. Trends Endocrinol Metab.

[99] Hirano I, Nakamura S, Yokota D, Ono T, Shigeno K, Fujisawa S, Shinjo K, Ohnishi K (2009). Depletion of Pleckstrin homology domain leucine-rich repeat protein phosphatases 1 and 2 by Bcr-Abl promotes chronic myelogenous leukemia cell proliferation through continuous phosphorylation of Akt isoforms. J Biol Chem.

[100] Xu Q, Simpson SE, Scialla TJ, Bagg A, Carroll M (2003). Survival of acute myeloid leukemia cells requires PI3 kinase activation. Blood.

[101] Min YH, Eom JI, Cheong JW, Maeng HO, Kim JY, Jeung HK, Lee ST, Lee MH, Hahn JS, Ko YW (2003). Constitutive phosphorylation of Akt/PKB protein in acute myeloid leukemia: its significance as a prognostic variable. Leukemia.

[102] Tazzari PL, Cappellini A, Grafone T, Mantovani I, Ricci F, Billi AM, Ottaviani E, Conte R, Martinelli G, Martelli AM (2004). Detection of serine 473 phosphorylated Akt in acute myeloid leukaemia blasts by flow cytometry. Br J Haematol.

[103] Grandage VL, Gale RE, Linch DC, Khwaja A (2005). PI3-kinase/Akt is constitutively active in primary acute myeloid leukaemia cells and regulates survival and chemoresistance via NF-κB, Mapkinase and p53 pathways. Leukemia.

[104] Gallay N, Dos Santos C, Cuzin L, Bousquet M, Simmonet Gouy V, Chaussade C, Attal M, Payrastre B, Demur C, Recher C (2009). The level of AKT phosphorylation on threonine 308 but not on serine 473 is associated with high-risk cytogenetics and predicts poor overall survival in acute myeloid leukaemia. Leukemia.

[105] Kornblau SM, Tibes R, Qiu YH, Chen W, Kantarjian HM, Andreeff M, Coombes KR, Mills GB (2009). Functional proteomic profiling of AML predicts response and survival. Blood.

[106] Bardet V, Tamburini J, Ifrah N, Dreyfus F, Mayeux P, Bouscary D, Lacombe C (2006). Single cell analysis of phosphoinositide 3-kinase/Akt and ERK activation in acute myeloid leukemia by flow cytometry. Haematologica.

[107] Recher C, Beyne-Rauzy O, Demur C, Chicanne G, Dos Santos C, Mas VM, Benzaquen D, Laurent G, Huguet F, Payrastre B (2005). Antileukemic activity of rapamycin in acute myeloid leukemia. Blood.

[108] Xu Q, Thompson JE, Carroll M (2005). mTOR regulates cell survival after etoposide treatment in primary AML cells. Blood.

[109] Billottet C, Grandage VL, Gale RE, Quattropani A, Rommel C, Vanhaesebroeck B, Khwaja A (2006). A selective inhibitor of the p110δ isoform of PI 3-kinase inhibits AML cell proliferation and survival and increases the cytotoxic effects of VP16. Oncogene.

[110] Doepfner KT, Spertini O, Arcaro A (2007). Autocrine insulin-like growth factor-I signaling promotes growth and survival of human acute myeloid leukemia cells via the phosphoinositide 3-kinase/Akt pathway. Leukemia.

[111] Billottet C, Banerjee L, Vanhaesebroeck B, Khwaja A (2009). Inhibition of class I phosphoinositide 3-kinase activity impairs proliferation and triggers apoptosis in acute promyelocytic leukemia without affecting atra-induced differentiation. Cancer Res.

[112] Tazzari PL, Tabellini G, Bortul R, Papa V, Evangelisti C, Grafone T, Martinelli G, McCubrey JA, Martelli AM (2007). The insulin-like growth factor-I receptor kinase inhibitor NVP-AEW541 induces apoptosis in acute myeloid leukemia cells exhibiting autocrine insulin-like growth factor-I secretion. Leukemia.

[113] Tamburini J, Chapuis N, Bardet V, Park S, Sujobert P, Willems L, Ifrah N, Dreyfus F, Mayeux P, Lacombe C, Bouscary D (2008). Mammalian target of rapamycin (mTOR) inhibition activates phosphatidylinositol 3-kinase/Akt by up-regulating insulin-like growth factor-1 receptor signaling in acute myeloid leukemia: rationale for therapeutic inhibition of both pathways. Blood.

[114] Chapuis N, Tamburini J, Cornillet-Lefebvre P, Gillot L, Bardet V, Willems L, Park S, Green AS, Ifrah N, Dreyfus F, Mayeux P, Lacombe C, Bouscary D (2010). Autocrine IGF-1/IGF-1R signaling is responsible for constitutive PI3K/Akt activation in acute myeloid leukemia: therapeutic value of neutralizing anti-IGF-1R antibody. Haematologica.

[115] Markovic A, MacKenzie KL, Lock RB (2005). FLT-3: a new focus in the understanding of acute leukemia. Int J Biochem Cell Biol.

[116] Tamburini J, Elie C, Bardet V, Chapuis N, Park S, Broet P, Cornillet-Lefebvre P, Lioure B, Ugo V, Blanchet O, Ifrah N, Witz F, Dreyfus F, Mayeux P, Lacombe C, Bouscary D (2007). Constitutive phosphoinositide 3-kinase/Akt activation represents a favorable prognostic factor in de novo acute myelogenous leukemia patients. Blood.

[117] Zatkova A, Schoch C, Speleman F, Poppe B, Mannhalter C, Fonatsch C, Wimmer K (2006). GAB2 is a novel target of 11q amplification in AML/MDS. Genes Chromosomes Cancer.

[118] Sun J, Pedersen M, Ronnstrand L (2008). Gab2 is involved in differential phosphoinositide 3-kinase signaling by two splice forms of c-Kit. J Biol Chem.

[119] Luo JM, Yoshida H, Komura S, Ohishi N, Pan L, Shigeno K, Hanamura I, Miura K, Iida S, Ueda R, Naoe T, Akao Y, Ohno R, Ohnishi K (2003). Possible dominant-negative mutation of the SHIP gene in acute myeloid leukemia. Leukemia.

[120] Cheong JW, Eom JI, Maeng HY, Lee ST, Hahn JS, Ko YW, Min YH (2003). Phosphatase and tensin homologue phosphorylation in the C-terminal regulatory domain is frequently observed in acute myeloid leukaemia and associated with poor clinical outcome. Br J Haematol.

[121] Kuo YC, Huang KY, Yang CH, Yang YS, Lee WY, Chiang CW (2008). Regulation of phosphorylation of Thr-308 of Akt, cell proliferation, and survival by the B55α regulatory subunit targeting of the protein phosphatase 2A holoenzyme to Akt. J Biol Chem.

[122] Ruvolo PP, Qui YH, Coombes KR, Zhang N, Ruvolo VR, Borthakur G, Konopleva M, Andreeff M, Kornblau SM (2011). Low expression of PP2A regulatory subunit B55α is associated with T308 phosphorylation of AKT and shorter complete remission duration in acute myeloid leukemia patients. Leukemia.

[123] Cristobal I, Garcia-Orti L, Cirauqui C, Alonso MM, Calasanz MJ, Odero MD (2011). PP2A impaired activity is a common event in acute myeloid leukemia and its activation by forskolin has a potent anti-leukemic effect. Leukemia.

[124] Cristobal I, Blanco FJ, Garcia-Orti L, Marcotegui N, Vicente C, Rifon J, Novo FJ, Bandres E, Calasanz MJ, Bernabeu C, Odero MD (2010). SETBP1 overexpression is a novel leukemogenic mechanism that predicts adverse outcome in elderly patients with acute myeloid leukemia. Blood.

[125] List AF, Glinsmann-Gibson B, Stadheim C, Meuillet EJ, Bellamy W, Powis G (2004). Vascular endothelial growth factor receptor-1 and receptor-2 initiate a phosphatidylinositide 3-kinase-dependent clonogenic response in acute myeloid leukemia cells. Expl Hematol.

[126] Wakabayashi M, Miwa H, Shikami M, Hiramatsu A, Ikai T, Tajima E, Yamamoto H, Miura K, Satoh A, Itoh M, Imamura A, Mihara H, Katoh Y, Nitta M (2004). Autocrine pathway of angiopoietins-Tie2 system in AML cells: association with phosphatidyl-inositol 3 kinase. Hematol J.

[127] Konopleva MY, Jordan CT (2011). Leukemia stem cells and microenvironment: biology and therapeutic targeting. J Clin Oncol.

[128] Matsunaga T, Takemoto N, Sato T, Takimoto R, Tanaka I, Fujimi A, Akiyama T, Kuroda H, Kawano Y, Kobune M, Kato J, Hirayama Y, Sakamaki S, Kohda K, Miyake K, Niitsu Y (2003). Interaction between leukemic-cell VLA-4 and stromal fibronectin is a decisive factor for minimal residual disease of acute myelogenous leukemia. Nat Med.

[129] Imai Y, Shimaoka M, Kurokawa M (2010). Essential roles of VLA-4 in the hematopoietic system. Int J Hematol.

[130] Tabe Y, Jin L, Tsutsumi-Ishii Y, Xu Y, McQueen T, Priebe W, Mills GB, Ohsaka A, Nagaoka I, Andreeff M, Konopleva M (2007). Activation of integrin-linked kinase is a critical prosurvival pathway induced in leukemic cells by bone marrow-derived stromal cells. Cancer Res.

[131] Tamburini J, Green AS, Bardet V, Chapuis N, Park S, Willems L, Uzunov M, Ifrah N, Dreyfus F, Lacombe C, Mayeux P, Bouscary D (2009). Protein synthesis is resistant to rapamycin and constitutes a promising therapeutic target in acute myeloid leukemia. Blood.

[132] Dos Santos C, Demur C, Bardet V, Prade-Houdellier N, Payrastre B, Recher C (2008). A critical role for Lyn in acute myeloid leukemia. Blood.

[133] Chow S, Minden MD, Hedley DW (2006). Constitutive phosphorylation of the S6 ribosomal protein via mTOR and ERK signaling in the peripheral blasts of acute leukemia patients. Exp Hematol.

[134] Ricciardi MR, McQueen T, Chism D, Milella M, Estey E, Kaldjian E, Sebolt-Leopold J, Konopleva M, Andreeff M (2005). Quantitative single cell determination of ERK phosphorylation and regulation in relapsed and refractory primary acute myeloid leukemia. Leukemia.

[135] Silva A, Yunes JA, Cardoso BA, Martins LR, Jotta PY, Abecasis M, Nowill AE, Leslie NR, Cardoso AA, Barata JT (2008). PTEN posttranslational inactivation and hyperactivation of the PI3K/Akt pathway sustain primary T cell leukemia viability. J Clin Invest.

[136] Jotta PY, Ganazza MA, Silva A, Viana MB, da Silva MJ, Zambaldi LJ, Barata JT, Brandalise SR, Yunes JA (2010). Negative prognostic impact of PTEN mutation in pediatric T-cell acute lymphoblastic leukemia. Leukemia.

[137] Chan SM, Weng AP, Tibshirani R, Aster JC, Utz PJ (2007). Notch signals positively regulate activity of the mTOR pathway in T-cell acute lymphoblastic leukemia. Blood.

[138] Palomero T, Sulis ML, Cortina M, Real PJ, Barnes K, Ciofani M, Caparros E, Buteau J, Brown K, Perkins SL, Bhagat G, Agarwal AM, Basso G, Castillo M, Nagase S, Cordon-Cardo C, Parsons R, Zuniga-Pflucker JC, Dominguez M, Ferrando AA (2007). Mutational loss of PTEN induces resistance to NOTCH1 inhibition in T-cell leukemia. Nat Med.

[139] Larson Gedman A, Chen Q, Kugel Desmoulin S, Ge Y, LaFiura K, Haska CL, Cherian C, Devidas M, Linda SB, Taub JW, Matherly LH (2009). The impact of NOTCH1, FBW7 and PTEN mutations on prognosis and downstream signaling in pediatric T-cell acute lymphoblastic leukemia: a report from the Children’s Oncology Group. Leukemia.

[140] Medyouf H, Gao X, Armstrong F, Gusscott S, Liu Q, Gedman AL, Matherly LH, Schultz KR, Pflumio F, You MJ, Weng AP (2010). Acute T-cell leukemias remain dependent on Notch signaling despite PTEN and INK4A/ARF loss. Blood.

[141] Mavrakis KJ, Wolfe AL, Oricchio E, Palomero T, de Keersmaecker K, McJunkin K, Zuber J, James T, Khan AA, Leslie CS, Parker JS, Paddison PJ, Tam W, Ferrando A, Wendel HG (2010). Genome-wide RNA-mediated interference screen identifies miR-19 targets in Notch-induced T-cell acute lymphoblastic leukaemia. Nat Cell Biol.

[142] Palomero T, Lim WK, Odom DT, Sulis ML, Real PJ, Margolin A, Barnes KC, O’Neil J, Neuberg D, Weng AP, Aster JC, Sigaux F, Soulier J, Look AT, Young RA, Califano A, Ferrando AA (2006). NOTCH1 directly regulates c-MYC and activates a feed-forward-loop transcriptional network promoting leukemic cell growth. Proc Natl Acad Sci U S A.

[143] Gutierrez A, Grebliunaite R, Feng H, Kozakewich E, Zhu S, Guo F, Payne E, Mansour M, Dahlberg SE, Neuberg DS, den Hertog J, Prochownik EV, Testa JR, Harris M, Kanki JP, Look AT (2011). Pten mediates Myc oncogene dependence in a conditional zebrafish model of T cell acute lymphoblastic leukemia. J Exp Med.

[144] Gutierrez A, Sanda T, Grebliunaite R, Carracedo A, Salmena L, Ahn Y, Dahlberg S, Neuberg D, Moreau LA, Winter SS, Larson R, Zhang J, Protopopov A, Chin L, Pandolfi PP, Silverman LB, Hunger SP, Sallan SE, Look AT (2009). High frequency of PTEN, PI3K, and AKT abnormalities in T-cell acute lymphoblastic leukemia. Blood.

[145] Lo TC, Barnhill LM, Kim Y, Nakae EA, Yu AL, Diccianni MB (2009). Inactivation of SHIP1 in T-cell acute lymphoblastic leukemia due to mutation and extensive alternative splicing. Leuk Res.

[146] Medyouf H, Gusscott S, Wang H, Tseng JC, Wai C, Nemirovsky O, Trumpp A, Pflumio F, Carboni J, Gottardis M, Pollak M, Kung AL, Aster JC, Holzenberger M, Weng AP (2011). High-level IGF1R expression is required for leukemia-initiating cell activity in T-ALL and is supported by Notch signaling. J Exp Med.

[147] Cardoso BA, Martins LR, Santos CI, Nadler LM, Boussiotis VA, Cardoso AA, Barata JT (2009). Interleukin-4 stimulates proliferation and growth of T-cell acute lymphoblastic leukemia cells by activating mTOR signaling. Leukemia.

[148] Barata JT, Silva A, Brandao JG, Nadler LM, Cardoso AA, Boussiotis VA (2004). Activation of PI3K is indispensable for interleukin 7-mediated viability, proliferation, glucose use, and growth of T cell acute lymphoblastic leukemia cells. J Exp Med.

[149] Scupoli MT, Perbellini O, Krampera M, Vinante F, Cioffi F, Pizzolo G (2007). Interleukin 7 requirement for survival of T-cell acute lymphoblastic leukemia and human thymocytes on bone marrow stroma. Haematologica.

[150] Silva A, Girio A, Cebola I, Santos CI, Antunes F, Barata JT (2011). Intracellular reactive oxygen species are essential for PI3K/Akt/mTOR-dependent IL-7-mediated viability of T-cell acute lymphoblastic leukemia cells. Leukemia.

[151] Scupoli MT, Vinante F, Krampera M, Vincenzi C, Nadali G, Zampieri F, Ritter MA, Eren E, Santini F, Pizzolo G (2003). Thymic epithelial cells promote survival of human T-cell acute lymphoblastic leukemia blasts: the role of interleukin-7. Haematologica.

[152] Zenatti PP, Ribeiro D, Li W, Zuurbier L, Silva MC, Paganin M, Tritapoe J, Hixon JA, Silveira AB, Cardoso BA, Sarmento LM, Correia N, Toribio ML, Kobarg J, Horstmann M, Pieters R, Brandalise SR, Ferrando AA, Meijerink JP, Durum SK, Yunes JA, Barata JT (2011). Oncogenic IL7R gain-of-function mutations in childhood T-cell acute lymphoblastic leukemia. Nat Genet.

[153] Wong D, Korz W (2008). Translating an Antagonist of Chemokine Receptor CXCR4: from bench to bedside. Clin Cancer Res.

[154] Scupoli MT, Donadelli M, Cioffi F, Rossi M, Perbellini O, Malpeli G, Corbioli S, Vinante F, Krampera M, Palmieri M, Scarpa A, Ariola C, Foa R, Pizzolo G (2008). Bone marrow stromal cells and the upregulation of interleukin-8 production in human T-cell acute lymphoblastic leukemia through the CXCL12/CXCR4 axis and the NF-κB and JNK/AP-1 pathways. Haematologica.

[155] Pillozzi S, Masselli M, De Lorenzo E, Accordi B, Cilia E, Crociani O, Amedei A, Veltroni M, D’Amico M, Basso G, Becchetti A, Campana D, Arcangeli A (2011). Chemotherapy resistance in acute lymphoblastic leukemia requires hERG1 channels and is overcome by hERG1 blockers. Blood.

[156] Gregorj C, Ricciardi MR, Petrucci MT, Scerpa MC, De Cave F, Fazi P, Vignetti M, Vitale A, Mancini M, Cimino G, Palmieri S, Di Raimondo F, Specchia G, Fabbiano F, Cantore N, Mosna F, Camera A, Luppi M, Annino L, Miraglia E, Fioritoni G, Ronco F, Meloni G, Mandelli F, Andreeff M, Milella M, Foa R, Tafuri A (2007). ERK1/2 phosphorylation is an independent predictor of complete remission in newly diagnosed adult acute lymphoblastic leukemia. Blood.

[157] Sattler M, Salgia R, Okuda K, Uemura N, Durstin MA, Pisick E, Xu G, Li JL, Prasad KV, Griffin JD (1996). The proto-oncogene product p120CBL and the adaptor proteins CRKL and c-CRK link c-ABL, p190BCR/ABL and p210BCR/ABL to the phosphatidylinositol-3’ kinase pathway. Oncogene.

[158] Ly C, Arechiga AF, Melo JV, Walsh CM, Ong ST (2003). Bcr-Abl kinase modulates the translation regulators ribosomal protein S6 and 4E-BP1 in chronic myelogenous leukemia cells via the mammalian target of rapamycin. Cancer Res.

[159] Kim JH, Chu SC, Gramlich JL, Pride YB, Babendreier E, Chauhan D, Salgia R, Podar K, Griffin JD, Sattler M (2005). Activation of the PI3K/mTOR pathway by BCR-ABL contributes to increased production of reactive oxygen species. Blood.

[160] Kharas MG, Fruman DA (2005). ABL oncogenes and phosphoinositide 3-kinase: mechanism of activation and downstream effectors. Cancer Res.

[161] Redig AJ, Vakana E, Platanias LC (2011). Regulation of mammalian target of rapamycin and mitogen activated protein kinase pathways by BCR-ABL. Leuk Lymphoma.

[162] Ren SY, Xue F, Feng J, Skorski T (2005). Intrinsic regulation of the interactions between the SH3 domain of p85 subunit of phosphatidylinositol-3 kinase and the protein network of BCR/ABL oncogenic tyrosine kinase. Exp Hematol.

[163] Harrison-Findik D, Susa M, Varticovski L (1995). Association of phosphatidylinositol 3-kinase with SHC in chronic myelogeneous leukemia cells. Oncogene.

[164] Parmar S, Smith J, Sassano A, Uddin S, Katsoulidis E, Majchrzak B, Kambhampati S, Eklund EA, Tallman MS, Fish EN, Platanias LC (2005). Differential regulation of the p70 S6 kinase pathway by interferon alpha (IFNα) and imatinib mesylate (STI571) in chronic myelogenous leukemia cells. Blood.

[165] Hirase C, Maeda Y, Takai S, Kanamaru A (2009). Hypersensitivity of Ph-positive lymphoid cell lines to rapamycin: Possible clinical application of mTOR inhibitor. Leuk Res.

[166] Kharas MG, Janes MR, Scarfone VM, Lilly MB, Knight ZA, Shokat KM, Fruman DA (2008). Ablation of PI3K blocks BCR-ABL leukemogenesis in mice, and a dual PI3K/mTOR inhibitor prevents expansion of human BCR-ABL+ leukemia cells. J Clin Invest.

[167] Martin KA, Schalm SS, Romanelli A, Keon KL, Blenis J (2001). Ribosomal S6 kinase 2 inhibition by a potent C-terminal repressor domain is relieved by mitogen-activated protein-extracellular signal-regulated kinase kinase-regulated phosphorylation. J Biol Chem.

[168] Wlodarski P, Kasprzycka M, Liu X, Marzec M, Robertson ES, Slupianek A, Wasik MA (2005). Activation of mammalian target of rapamycin in transformed B lymphocytes is nutrient dependent but independent of Akt, mitogen-activated protein kinase/extracellular signal-regulated kinase kinase, insulin growth factor-I, and serum. Cancer Res.

[169] Gulati P, Thomas G (2007). Nutrient sensing in the mTOR/S6K1 signalling pathway. Biochem Soc Trans.

[170] Quentmeier H, Eberth S, Romani J, Zaborski M, Drexler HG (2011). BCR-ABL1-independent PI3Kinase activation causing imatinib-resistance. J Hematol Oncol.

[171] Neviani P, Santhanam R, Trotta R, Notari M, Blaser BW, Liu S, Mao H, Chang JS, Galietta A, Uttam A, Roy DC, Valtieri M, Bruner-Klisovic R, Caligiuri MA, Bloomfield CD, Marcucci G, Perrotti D (2005). The tumor suppressor PP2A is functionally inactivated in blast crisis CML through the inhibitory activity of the BCR/ABL-regulated SET protein. Cancer Cell.

[172] Killestein J, Rudick RA, Polman CH (2011). Oral treatment for multiple sclerosis. Lancet Neurol.

[173] Neviani P, Santhanam R, Oaks JJ, Eiring AM, Notari M, Blaser BW, Liu S, Trotta R, Muthusamy N, Gambacorti-Passerini C, Druker BJ, Cortes J, Marcucci G, Chen CS, Verrills NM, Roy DC, Caligiuri MA, Bloomfield CD, Byrd JC, Perrotti D (2007). FTY720, a new alternative for treating blast crisis chronic myelogenous leukemia and Philadelphia chromosome-positive acute lymphocytic leukemia. J Clin Invest.

[174] Brown VI, Fang J, Alcorn K, Barr R, Kim JM, Wasserman R, Grupp SA (2003). Rapamycin is active against B-precursor leukemia in vitro and in vivo, an effect that is modulated by IL-7-mediated signaling. Proc Natl Acad Sci USA.

[175] Wang L, Fortney JE, Gibson LF (2004). Stromal cell protection of B-lineage acute lymphoblastic leukemic cells during chemotherapy requires active Akt. Leuk Res.

[176] Bertrand FE, Spengemen JD, Shelton JG, McCubrey JA (2005). Inhibition of PI3K, mTOR and MEK signaling pathways promotes rapid apoptosis in B-lineage ALL in the presence of stromal cell support. Leukemia.

[177] Brown VI, Hulitt J, Fish J, Sheen C, Bruno M, Xu Q, Carroll M, Fang J, Teachey D, Grupp SA (2007). Thymic stromal-derived lymphopoietin induces proliferation of pre-B leukemia and antagonizes mTOR inhibitors, suggesting a role for interleukin-7Ralpha signaling. Cancer Res.

[178] Juarez J, Baraz R, Gaundar S, Bradstock K, Bendall L (2007). Interaction of interleukin-7 and interleukin-3 with the CXCL12-induced proliferation of B-cell progenitor acute lymphoblastic leukemia. Haematologica.

[179] Shalapour S, Hof J, Kirschner-Schwabe R, Bastian L, Eckert C, Prada J, Henze G, von Stackelberg A, Seeger K (2011). High VLA-4 expression is associated with adverse outcome and distinct gene expression changes in childhood B-cell precursor acute lymphoblastic leukemia at first relapse. Haematologica.

[180] Shochat C, Tal N, Bandapalli OR, Palmi C, Ganmore I, te Kronnie G, Cario G, Cazzaniga G, Kulozik AE, Stanulla M, Schrappe M, Biondi A, Basso G, Bercovich D, Muckenthaler MU, Izraeli S (2011). Gain-of-function mutations in interleukin-7 receptor-α (IL7R) in childhood acute lymphoblastic leukemias. J Exp Med.

[181] Fuka G, Kantner HP, Grausenburger R, Inthal A, Bauer E, Krapf G, Kaindl U, Kauer M, Dworzak MN, Stoiber D, Haas OA, Panzer-Grumayer R (2011). Silencing of ETV6/RUNX1 abrogates PI3K/AKT/mTOR signaling and impairs reconstitution of leukemia in xenografts. Leukemia.

[182] Zhang J, Grindley JC, Yin T, Jayasinghe S, He XC, Ross JT, Haug JS, Rupp D, Porter-Westpfahl KS, Wiedemann LM, Wu H, Li L (2006). PTEN maintains haematopoietic stem cells and acts in lineage choice and leukaemia prevention. Nature.

[183] Yilmaz OH, Valdez R, Theisen BK, Guo W, Ferguson DO, Wu H, Morrison SJ (2006). Pten dependence distinguishes haematopoietic stem cells from leukaemia-initiating cells. Nature.

[184] Guo W, Schubbert S, Chen JY, Valamehr B, Mosessian S, Shi H, Dang NH, Garcia C, Theodoro MF, Varella-Garcia M, Wu H (2011). Suppression of leukemia development caused by PTEN loss. Proc Natl Acad Sci USA.

[185] Guo W, Lasky JL, Chang CJ, Mosessian S, Lewis X, Xiao Y, Yeh JE, Chen JY, Iruela-Arispe ML, Varella-Garcia M, Wu H (2008). Multi-genetic events collaboratively contribute to Pten-null leukaemia stem-cell formation. Nature.

[186] Yu H, Li Y, Gao C, Fabien L, Jia Y, Lu J, Silberstein LE, Pinkus GS, Ye K, Chai L, Luo HR (2010). Relevant mouse model for human monocytic leukemia through Cre/lox-controlled myeloid-specific deletion of PTEN. Leukemia.

[187] Peng C, Chen Y, Yang Z, Zhang H, Osterby L, Rosmarin AG, Li S (2010). PTEN is a tumor suppressor in CML stem cells and BCR-ABL-induced leukemias in mice. Blood.

[188] Munugalavadla V, Sims EC, Borneo J, Chan RJ, Kapur R (2007). Genetic and pharmacologic evidence implicating the p85 α, but not p85 β, regulatory subunit of PI3K and Rac2 GTPase in regulating oncogenic KIT-induced transformation in acute myeloid leukemia and systemic mastocytosis. Blood.

[189] Wymann MP, Bulgarelli-Leva G, Zvelebil MJ, Pirola L, Vanhaesebroeck B, Waterfield MD, Panayotou G (1996). Wortmannin inactivates phosphoinositide 3-kinase by covalent modification of Lys-802, a residue involved in the phosphate transfer reaction. Mol Cell Biol.

[190] Vlahos CJ, Matter WF, Hui KY, Brown RF (1994). A specific inhibitor of phosphatidylinositol 3-kinase, 2-(4-morpholinyl)-8-phenyl-4H-1-benzopyran-4-one (LY294002). J Biol Chem.

[191] Garcia-Echeverria C, Sellers WR (2008). Drug discovery approaches targeting the PI3K/Akt pathway in cancer. Oncogene.

[192] O’Gorman DM, McKenna SL, McGahon AJ, Knox KA, Cotter TG (2000). Sensitisation of HL60 human leukaemic cells to cytotoxic drug-induced apoptosis by inhibition of PI3-kinase survival signals. Leukemia.

[193] Neri LM, Borgatti P, Tazzari PL, Bortul R, Cappellini A, Tabellini G, Bellacosa A, Capitani S, Martelli AM (2003). The phosphoinositide 3-kinase/AKT1 pathway involvement in drug and all-trans-retinoic acid resistance of leukemia cells. Mol Cancer Res.

[194] Zhao S, Konopleva M, Cabreira-Hansen M, Xie Z, Hu W, Milella M, Estrov Z, Mills GB, Andreeff M (2004). Inhibition of phosphatidylinositol 3-kinase dephosphorylates BAD and promotes apoptosis in myeloid leukemias. Leukemia.

[195] Uddin S, Hussain A, Al-Hussein K, Platanias LC, Bhatia KG (2004). Inhibition of phosphatidylinositol 3’-kinase induces preferentially killing of PTEN-null T leukemias through AKT pathway. Biochem Biophys Res Commun.

[196] Sujobert P, Bardet V, Cornillet-Lefebvre P, Hayflick JS, Prie N, Verdier F, Vanhaesebroeck B, Muller O, Pesce F, Ifrah N, Hunault-Berger M, Berthou C, Villemagne B, Jourdan E, Audhuy B, Solary E, Witz B, Harousseau JL, Himberlin C, Lamy T, Lioure B, Cahn JY, Dreyfus F, Mayeux P, Lacombe C, Bouscary D (2005). Essential role for the p110δ isoform in phosphoinositide 3-kinase activation and cell proliferation in acute myeloid leukemia. Blood.

[197] Lannutti BJ, Meadows SA, Herman SE, Kashishian A, Steiner B, Johnson AJ, Byrd JC, Tyner JW, Loriaux MM, Deininger M, Druker BJ, Puri KD, Ulrich RG, Giese NA (2011). CAL-101, a p110δ selective phosphatidylinositol-3-kinase inhibitor for the treatment of B-cell malignancies, inhibits PI3K signaling and cellular viability. Blood.

[198] Herman SE, Lapalombella R, Gordon AL, Ramanunni A, Blum KA, Jones J, Zhang X, Lannutti BJ, Puri KD, Muthusamy N, Byrd JC, Johnson AJ (2011). The role of phosphatidylinositol 3-kinase-δ in the immunomodulatory effects of lenalidomide in chronic lymphocytic leukemia. Blood.

[199] Avellino R, Romano S, Parasole R, Bisogni R, Lamberti A, Poggi V, Venuta S, Romano MF (2005). Rapamycin stimulates apoptosis of childhood acute lymphoblastic leukemia cells. Blood.

[200] Teachey DT, Obzut DA, Cooperman J, Fang J, Carroll M, Choi JK, Houghton PJ, Brown VI, Grupp SA (2006). The mTOR inhibitor CCI-779 induces apoptosis and inhibits growth in preclinical models of primary adult human ALL. Blood.

[201] Zeng Z, Sarbassov dos D, Samudio IJ, Yee KW, Munsell MF, Ellen Jackson C, Giles FJ, Sabatini DM, Andreeff M, Konopleva M (2007). Rapamycin derivatives reduce mTORC2 signaling and inhibit AKT activation in AML. Blood.

[202] Crazzolara R, Bradstock KF, Bendall LJ (2009). RAD001 (Everolimus) induces autophagy in acute lymphoblastic leukemia. Autophagy.

[203] Bonapace L, Bornhauser BC, Schmitz M, Cario G, Ziegler U, Niggli FK, Schafer BW, Schrappe M, Stanulla M, Bourquin JP (2010). Induction of autophagy-dependent necroptosis is required for childhood acute lymphoblastic leukemia cells to overcome glucocorticoid resistance. J Clin Invest.

[204] Batista A, Barata JT, Raderschall E, Sallan SE, Carlesso N, Nadler LM, Cardoso AA (2011). Targeting of active mTOR inhibits primary leukemia T cells and synergizes with cytotoxic drugs and signaling inhibitors. Exp Hematol.

[205] Wei G, Twomey D, Lamb J, Schlis K, Agarwal J, Stam RW, Opferman JT, Sallan SE, den Boer ML, Pieters R, Golub TR, Armstrong SA (2006). Gene expression-based chemical genomics identifies rapamycin as a modulator of MCL1 and glucocorticoid resistance. Cancer Cell.

[206] Gu L, Zhou C, Liu H, Gao J, Li Q, Mu D, Ma Z (2010). Rapamycin sensitizes T-ALL cells to dexamethasone-induced apoptosis. J Exp Clin Cancer Res.

[207] Nishioka C, Ikezoe T, Yang J, Koeffler HP, Yokoyama A (2008). Blockade of mTOR signaling potentiates the ability of histone deacetylase inhibitor to induce growth arrest and differentiation of acute myelogenous leukemia cells. Leukemia.

[208] Saunders P, Cisterne A, Weiss J, Bradstock KF, Bendall LJ (2011). The mammalian target of rapamycin inhibitor RAD001 (everolimus) synergizes with chemotherapeutic agents, ionizing radiation and proteasome inhibitors in pre-B acute lymphocytic leukemia. Haematologica.

[209] Tabellini G, Tazzari PL, Bortul R, Evangelisti C, Billi AM, Grafone T, Martinelli G, Baccarani M, Martelli AM (2005). Phosphoinositide 3-kinase/Akt inhibition increases arsenic trioxide-induced apoptosis of acute promyelocytic and T-cell leukaemias. Br J Haematol.

[210] Tabellini G, Cappellini A, Tazzari PL, Falà F, Billi AM, Manzoli L, Cocco L, Martelli AM (2005). Phosphoinositide 3-kinase/Akt involvement in arsenic trioxide resistance of human leukemia cells. J Cell Physiol.

[211] Follo MY, Mongiorgi S, Bosi C, Cappellini A, Finelli C, Chiarini F, Papa V, Libra M, Martinelli G, Cocco L, Martelli AM (2007). The Akt/mammalian target of rapamycin signal transduction pathway is activated in high-risk myelodysplastic syndromes and influences cell survival and proliferation. Cancer Res.

[212] Yee KW, Zeng Z, Konopleva M, Verstovsek S, Ravandi F, Ferrajoli A, Thomas D, Wierda W, Apostolidou E, Albitar M, O’Brien S, Andreeff M, Giles FJ (2006). Phase I/II study of the mammalian target of rapamycin inhibitor everolimus (RAD001) in patients with relapsed or refractory hematologic malignancies. Clin Cancer Res.

[213] Rizzieri DA, Feldman E, Dipersio JF, Gabrail N, Stock W, Strair R, Rivera VM, Albitar M, Bedrosian CL, Giles FJ (2008). A phase 2 clinical trial of deforolimus (AP23573, MK-8669), a novel mammalian target of rapamycin inhibitor, in patients with relapsed or refractory hematologic malignancies. Clin Cancer Res.

[214] Boehm A, Mayerhofer M, Herndlhofer S, Knoebl P, Sillaber C, Sperr WR, Jaeger U, Valent P (2009). Evaluation of in vivo antineoplastic effects of rapamycin in patients with chemotherapy-refractory AML. Eur J Intern Med.

[215] Fan QW, Knight ZA, Goldenberg DD, Yu W, Mostov KE, Stokoe D, Shokat KM, Weiss WA (2006). A dual PI3 kinase/mTOR inhibitor reveals emergent efficacy in glioma. Cancer Cell.

[216] Fan QW, Cheng CK, Nicolaides TP, Hackett CS, Knight ZA, Shokat KM, Weiss WA (2007). A dual phosphoinositide-3-kinase α/mTOR inhibitor cooperates with blockade of epidermal growth factor receptor in PTEN-mutant glioma. Cancer Res.

[217] Maira SM, Stauffer F, Brueggen J, Furet P, Schnell C, Fritsch C, Brachmann S, Chene P, De Pover A, Schoemaker K, Fabbro D, Gabriel D, Simonen M, Murphy L, Finan P, Sellers W, Garcia-Echeverria C (2008). Identification and characterization of NVP-BEZ235, a new orally available dual phosphatidylinositol 3-kinase/mammalian target of rapamycin inhibitor with potent in vivo antitumor activity. Mol Cancer Ther.

[218] Prasad G, Sottero T, Yang X, Mueller S, James CD, Weiss WA, Polley MY, Ozawa T, Berger MS, Aftab DT, Prados MD, Haas-Kogan DA (2011). Inhibition of PI3K/mTOR pathways in glioblastoma and implications for combination therapy with temozolomide. Neuro Oncol.

[219] Mallon R, Feldberg LR, Lucas J, Chaudhary I, Dehnhardt C, Santos ED, Chen Z, dos Santos O, Ayral-Kaloustian S, Venkatesan A, Hollander I (2011). Antitumor efficacy of PKI-587, a highly potent dual PI3K/mTOR kinase inhibitor. Clin Cancer Res.

[220] Yuan J, Mehta PP, Yin MJ, Sun S, Zou A, Chen J, Rafidi K, Feng Z, Nickel J, Engebretsen J, Hallin J, Blasina A, Zhang E, Nguyen L, Sun M, Vogt PK, McHarg A, Cheng H, Christensen JG, Kan JL, Bagrodia S (2011). PF-04691502, a Potent and Selective Oral Inhibitor of PI3K and mTOR Kinases with Antitumor Activity. Mol Cancer Ther.

[221] Li T, Wang J, Wang X, Yang N, Chen SM, Tong LJ, Yang CH, Meng LH, Ding J (2010). WJD008, a dual phosphatidylinositol 3-kinase (PI3K)/mammalian target of rapamycin inhibitor, prevents PI3K signaling and inhibits the proliferation of transformed cells with oncogenic PI3K mutant. J Pharmacol Exp Ther.

[222] Mallon R, Hollander I, Feldberg L, Lucas J, Soloveva V, Venkatesan A, Dehnhardt C, Delos Santos E, Chen Z, Dos Santos O, Ayral-Kaloustian S, Gibbons J (2010). Antitumor efficacy profile of PKI-402, a dual phosphatidylinositol 3-kinase/mammalian target of rapamycin inhibitor. Mol Cancer Ther.

[223] Heffron TP, Berry M, Castanedo G, Chang C, Chuckowree I, Dotson J, Folkes A, Gunzner J, Lesnick JD, Lewis C, Mathieu S, Nonomiya J, Olivero A, Pang J, Peterson D, Salphati L, Sampath D, Sideris S, Sutherlin DP, Tsui V, Wan NC, Wang S, Wong S, Zhu BY (2010). Identification of GNE-477, a potent and efficacious dual PI3K/mTOR inhibitor. Bioorg Med Chem Lett.

[224] Brachmann S, Fritsch C, Maira SM, Garcia-Echeverria C (2009). PI3K and mTOR inhibitors: a new generation of targeted anticancer agents. Curr Opin Cell Biol.

[225] Kojima K, Shimanuki M, Shikami M, Samudio IJ, Ruvolo V, Corn P, Hanaoka N, Konopleva M, Andreeff M, Nakakuma H (2008). The dual PI3 kinase/mTOR inhibitor PI-103 prevents p53 induction by Mdm2 inhibition but enhances p53-mediated mitochondrial apoptosis in p53 wild-type AML. Leukemia.

[226] Shangary S, Wang S (2009). Small-molecule inhibitors of the MDM2-p53 protein-protein interaction to reactivate p53 function: a novel approach for cancer therapy. Annu Rev Pharmacol Toxicol.

[227] Ploner C, Kofler R, Villunger A (2008). Noxa: at the tip of the balance between life and death. Oncogene.

[228] Park S, Chapuis N, Bardet V, Tamburini J, Gallay N, Willems L, Knight ZA, Shokat KM, Azar N, Viguie F, Ifrah N, Dreyfus F, Mayeux P, Lacombe C, Bouscary D (2008). PI-103, a dual inhibitor of Class IA phosphatidylinositide 3-kinase and mTOR, has antileukemic activity in AML. Leukemia.

[229] Hong Z, Xiao M, Yang Y, Han Z, Cao Y, Li C, Wu Y, Gong Q, Zhou X, Xu D, Meng L, Ma D, Zhou J (2011). Arsenic disulfide synergizes with the phosphoinositide 3-kinase inhibitor PI-103 to eradicate acute myeloid leukemia stem cells by inducing differentiation. Carcinogenesis.

[230] Emadi A, Gore SD (2010). Arsenic trioxide - An old drug rediscovered. Blood Rev.

[231] Chiarini F, Falà F, Tazzari PL, Ricci F, Astolfi A, Pession A, Pagliaro P, McCubrey JA, Martelli AM (2009). Dual inhibition of class IA phosphatidylinositol 3-kinase and mammalian target of rapamycin as a new therapeutic option for T-cell acute lymphoblastic leukemia. Cancer Res.

[232] Evangelisti C, Ricci F, Tazzari P, Tabellini G, Battistelli M, Falcieri E, Chiarini F, Bortul R, Melchionda F, Pagliaro P, Pession A, McCubrey JA, Martelli AM (2011). Targeted inhibition of mTORC1 and mTORC2 by active-site mTOR inhibitors has cytotoxic effects in T-cell acute lymphoblastic leukemia. Leukemia.

[233] Silverman LB, Stevenson KE, O’Brien JE, Asselin BL, Barr RD, Clavell L, Cole PD, Kelly KM, Laverdiere C, Michon B, Schorin MA, Schwartz CL, O’Holleran EW, Neuberg DS, Cohen HJ, Sallan SE (2010). Long-term results of Dana-Farber Cancer Institute ALL Consortium protocols for children with newly diagnosed acute lymphoblastic leukemia (1985-2000). Leukemia.

[234] Janes MR, Limon JJ, So L, Chen J, Lim RJ, Chavez MA, Vu C, Lilly MB, Mallya S, Ong ST, Konopleva M, Martin MB, Ren P, Liu Y, Rommel C, Fruman DA (2010). Effective and selective targeting of leukemia cells using a TORC1/2 kinase inhibitor. Nature Med.

[235] Raynaud FI, Eccles S, Clarke PA, Hayes A, Nutley B, Alix S, Henley A, Di-Stefano F, Ahmad Z, Guillard S, Bjerke LM, Kelland L, Valenti M, Patterson L, Gowan S, de Haven Brandon A, Hayakawa M, Kaizawa H, Koizumi T, Ohishi T, Patel S, Saghir N, Parker P, Waterfield M, Workman P (2007). Pharmacologic characterization of a potent inhibitor of class I phosphatidylinositide 3-kinases. Cancer Res.

[236] Chapuis N, Tamburini J, Green AS, Vignon C, Bardet V, Neyret A, Pannetier M, Willems L, Park S, Macone A, Maira SM, Ifrah N, Dreyfus F, Herault O, Lacombe C, Mayeux P, Bouscary D (2010). Dual inhibition of PI3K and mTORC1/2 signaling by NVP-BEZ235 as a new therapeutic strategy for acute myeloid leukemia. Clin Cancer Res.

[237] Jacinto E, Loewith R, Schmidt A, Lin S, Ruegg MA, Hall A, Hall MN (2004). Mammalian TOR complex 2 controls the actin cytoskeleton and is rapamycin insensitive. Nat Cell Biol.

[238] Clemens MJ (2004). Targets and mechanisms for the regulation of translation in malignant transformation. Oncogene.

[239] Chiarini F, Grimaldi C, Ricci F, Tazzari PL, Evangelisti C, Ognibene A, Battistelli M, Falcieri E, Melchionda F, Pession A, Pagliaro P, McCubrey JA, Martelli AM (2010). Activity of the novel dual phosphatidylinositol 3-kinase/mammalian target of rapamycin inhibitor NVP-BEZ235 against T-cell acute lymphoblastic leukemia. Cancer Res.

[240] Konopleva M, Konoplev S, Hu W, Zaritskey AY, Afanasiev BV, Andreeff M (2002). Stromal cells prevent apoptosis of AML cells by up-regulation of anti-apoptotic proteins. Leukemia.

[241] Schult C, Dahlhaus M, Glass A, Fischer K, Lange S, Freund M, Junghanss C (2012). The dual kinase inhibitor NVP-BEZ235 in combination with cytotoxic drugs exerts anti-proliferative activity towards acute lymphoblastic leukemia cells. Anticancer Res.

[242] Schuster K, Zheng J, Arbini AA, Zhang CC, Scaglioni PP (2011). Selective targeting of the mTORC1/2 protein kinase complexes leads to antileukemic effects in vitro and in vivo. Blood Cancer J.

[243] Carracedo A, Ma L, Teruya-Feldstein J, Rojo F, Salmena L, Alimonti A, Egia A, Sasaki AT, Thomas G, Kozma SC, Papa A, Nardella C, Cantley LC, Baselga J, Pandolfi PP (2008). Inhibition of mTORC1 leads to MAPK pathway activation through a PI3K-dependent feedback loop in human cancer. J Clin Invest.

[244] Weisberg E, Banerji L, Wright RD, Barrett R, Ray A, Moreno D, Catley L, Jiang J, Hall-Meyers E, Sauveur-Michel M, Stone R, Galinsky I, Fox E, Kung AL, Griffin JD (2008). Potentiation of antileukemic therapies by the dual PI3K/PDK-1 inhibitor, BAG956: effects on BCR-ABL- and mutant FLT3-expressing cells. Blood.

[245] Marone R, Erhart D, Mertz AC, Bohnacker T, Schnell C, Cmiljanovic V, Stauffer F, Garcia-Echeverria C, Giese B, Maira SM, Wymann MP (2009). Targeting melanoma with dual phosphoinositide 3-kinase/mammalian target of rapamycin inhibitors. Mol Cancer Res.

[246] Janes MR, Fruman DA (2010). Targeting TOR dependence in cancer. Oncotarget.

[247] Zawel L (2010). P3Kα: A Driver of Tumor Metastasis?. Oncotarget.

[248] Chappell WH, Steelman LS, Long JM, Kempf RC, Abrams SL, Franklin RA, Bäsecke J, Stivala S, Donia M, Fagone P, Malaponte G, Mazzarino MC, Nicoletti F, Libra M, Maksimovic-Ivanic D, Mijatovic S, Montalto G, Cervello M, Laidler P, Milella M, Tafuri A, Bonati A, Evangelisti C, Cocco L, Martelli AM, McCubrey JA (2011). Ras/Raf/MEK/ERK and PI3K/PTEN/Akt/mTOR Inhibitors: Rationale and Importance to Inhibiting These Pathways in Human Health. Oncotarget.

[249] Agoulnik IU, Hodgson MC, Bowden WA, Ittmann MM (2011). INPP4B: the New Kid on the PI3K Block. Oncotarget.

[250] Adams J, Schachter NF, Liu JC, Zacksenhaus E, Egan SE (2011). Elevated PI3K signaling drives multiple Breast Cancer subtypes. Oncotarget.

[251] Sokolosky ML, Stadelman KM, Chappell WH, Abrams SL, Martelli AM, Stivala F, Libra M, Nicoletti F, Drobot LB, Franklin RA, Steelman LS, McCubrey JA (2011). Involvement of Akt-1 and mTOR in Sensitivity of Breast Cancer to Targeted Therapy. Oncotarget.

[252] Garrett JT, Chakrabarty A, Arteaga CL (2011). Will PI3K pathway inhibitors be effective as single agents in patients with cancer?. Oncotarget.

[253] Platanias L, Vakana E (2011). AMPK in BCR-ABL expressing leukemias. Regulatory effects and therapeutic implications. Oncotarget.

[254] Markman B, Dienstmann R, Tabernero J (2010). Targeting the PI3K/Akt/mTOR Pathway — Beyond Rapalogs. Oncotarget.

[255] Sacco A, Roccaro A, Ghobrial IM (2010). Role of dual PI3/Akt and mTOR inhibition in Waldenstrom’s Macroglobulinemia. Oncotarget.

[256] Altman JK, Sassano A, Platanias LC (2011). Targeting mTOR for the treatment of AML. New agents and new directions. Oncotarget.

[257] Schatz JH, Wendel HG (2011). Targeted cancer therapy: What if the driver is just a messenger?. Cell Cycle.

[258] Malina A, Cencic R, Pelletier R (2011). Targeting Translation Dependence in Cancer. Oncotarget.

[259] Zitzmann K, Ruden J, Brand S, Goke B, Lichtl J, Spottl G, Auernhammer CJ (2010). Compensatory activation of Akt in response to mTOR and Raf inhibitors - a rationale for dual-targeted therapy approaches in neuroendocrine tumor disease. Cancer Lett.

[260] Sunayama J, Matsuda K, Sato A, Tachibana K, Suzuki K, Narita Y, Shibui S, Sakurada K, Kayama T, Tomiyama A, Kitanaka C (2010). Crosstalk between the PI3K/mTOR and MEK/ERK pathways involved in the maintenance of self-renewal and tumorigenicity of glioblastoma stem-like cells. Stem Cells.

[261] Fruman DA, Bismuth G (2009). Fine tuning the immune response with PI3K. Immunol Rev.

[262] Peng C, Chen Y, Li D, Li S (2010). Role of Pten in leukemia stem cells. Oncotarget.

[263] Di J, Duiveman-de Boer T, Figdor CG, Torensma R (2011). Eradicating cancer cells: struggle with a chameleon. Oncotarget.

[264] Chomel JC, Turhan AG (2011). Chronic myeloid leukemia stem cells in the era of targeted therapies: resistance, persistence and long-term dormancy. Oncotarget.

[265] Lopez-Fauqued M, Gil R, Grueso J, Hernandez-Losa J, Pujol A, Moline T, Recio JA (2010). The dual PI3K/mTOR inhibitor PI-103 promotes immunosuppression, in vivo tumor growth and increases survival of sorafenib-treated melanoma cells. Int J Cancer.

[266] Diamandis M, White NM, Yousef GM (2010). Personalized medicine: marking a new epoch in cancer patient management. Mol Cancer Res.

[267] Lee M, Theodoropoulou M, Graw J, Roncaroli F, Zatelli MC, Pellegata NS (2011). Levels of p27 sensitize to dual PI3K/mTOR inhibition. Mol Cancer Ther.

[268] Ilic N, Utermark T, Widlund HR, Roberts TM (2011). PI3K-targeted therapy can be evaded by gene amplification along the MYC-eukaryotic translation initiation factor 4E (eIF4E) axis. Proc Natl Acad Sci USA.

[269] Palomero T, Ferrando A (2008). Oncogenic NOTCH1 control of MYC and PI3K: challenges and opportunities for anti-NOTCH1 therapy in T-cell acute lymphoblastic leukemias and lymphomas. Clin Cancer Res.

[270] Green AS, Grabar S, Tulliez M, Park S, Al-Nawakil C, Chapuis N, Jacque N, Willems L, Azar N, Ifrah N, Dreyfus F, Lacombe C, Mayeux P, Bouscary D, Tamburini J (2012). The eukaryotic Initiating Factor 4E protein is overexpressed, but its level has no prognostic impact in acute myeloid leukaemia. Br J Haematol.

[271] Zhao C, Vollrath D (2011). mTOR pathway activation in age-related retinal disease. Aging.

[272] Major P (2011). Potential of mTOR inhibitors for the treatment of subependymal giant cell astrocytomas in tuberous sclerosis complex. Aging.

[273] Steelman LS, Chappell WH, Abrams SL, Kempf RC, Long J, Laidler P, Mijatovic S, Maksimovic-Ivanic D, Stivala F, Mazzarino MC, Donia M, Fagone P, Malaponte G, Nicoletti F, Libra M, Milella M, Tafuri A, Bonati A, Bäsecke J, Cocco L, Evangelisti C, Martelli AM, Montalto G, Cervello M, McCubrey JA (2011). Roles of the Raf/MEK/ERK and PI3K/PTEN/Akt/mTOR pathways in controlling growth and sensitivity to therapy-implications for cancer and aging. Aging.

[274] Williamson DL (2011). Normalizing a hyperactive mTOR initiates muscle growth during obesity. Aging.

[275] Galluzzi L, Kepp O, Kroemer G (2010). TP53 and MTOR crosstalk to regulate cellular senescence. Aging.

[276] Korotchkina LG, Leontieva OV, Bukreeva EI, Demidenko ZN, Gudkov AV, Blagosklonny MV (2010). The choice between p53-induced senescence and quiescence is determined in part by the mTOR pathway. Aging.

